# Sex Differences in the Immune System Become Evident in the Perinatal Period in the Four Core Genotypes Mouse

**DOI:** 10.3389/fendo.2021.582614

**Published:** 2021-05-27

**Authors:** Mrinal K. Ghosh, Kuan-hui E. Chen, Riva Dill-Garlow, Lisa J. Ma, Tomohiro Yonezawa, Yuichiro Itoh, Lorena Rivera, Kelly C. Radecki, Quiming P. Wu, Arthur P. Arnold, H. Konrad Muller, Ameae M. Walker

**Affiliations:** ^1^ Division of Biomedical Sciences, School of Medicine, University of California, Riverside, Riverside, CA, United States; ^2^ Department of Integrative Biology & Physiology, University of California, Los Angeles, Los Angeles, CA, United States; ^3^ School of Medicine, University of Tasmania, Hobart, TAS, Australia

**Keywords:** immune sexual dimorphism, T cell development, perinatal testosterone, intra-thymic estradiol, S1PR1, Sry, thymic epithelium/stroma

## Abstract

We have used the four core genotypes (FCG) mouse model, which allows a distinction between effects of gonadal secretions and chromosomal complement, to determine when sex differences in the immune system first appear and what influences their development. Using splenic T cell number as a measure that could be applied to neonates with as yet immature immune responses, we found no differences among the four genotypes at postnatal day 1, but by day 7, clear sex differences were observed. These sex differences were unexpectedly independent of chromosomal complement and similar in degree to gonadectomized FCG adults: both neonatal and gonadectomized adult females (XX and XY) showed 2-fold the number of CD4+ and 7-fold the number of CD8+ T cells *versus* their male (XX and XY) counterparts. Appearance of this long-lived sex difference between days 1 and 7 suggested a role for the male-specific perinatal surge of testicular testosterone. Interference with the testosterone surge significantly de-masculinized the male CD4+, but not CD8+ splenic profile. Treatment of neonates demonstrated elevated testosterone limited mature cell egress from the thymus, whereas estradiol reduced splenic T cell seeding in females. Neonatal male splenic epithelium/stroma expressed aromatase mRNA, suggesting capacity for splenic conversion of perinatal testosterone into estradiol in males, which, similar to administration of estradiol in females, would result in reduced splenic T cell seeding. These sex steroid effects affected both CD4+ and CD8+ cells and yet interference with the testosterone surge only significantly de-masculinized the splenic content of CD4+ cells. For CD8+ cells, male cells in the thymus were also found to express one third the density of sphingosine-1-phosphate thymic egress receptors per cell compared to female, a male characteristic most likely an indirect result of Sry expression. Interestingly, the data also support a previously unrecognized role for non-gonadal estradiol in the promotion of intra-thymic cell proliferation in neonates of both sexes. Microarray analysis suggested the thymic epithelium/stroma as the source of this hormone. We conclude that some immune sex differences appear long before puberty and more than one mechanism contributes to differential numbers and distribution of T cells.

## Introduction

Females have more robust immune responses after immunization and infection when compared with males [reviewed in (1)], but relatively little is known about the genesis of this physiological sex difference. Because many immune cells have receptors for gonadal steroids, analyses of sex differences in immune responses have largely focused on the reversible effects of gonadal steroids and their metabolites in adult animals [e.g (2–5)]. These studies have produced abundant evidence that gonadal steroids influence immune function. However, sex differences in immunity seem to be more complicated than simple regulation by postpubertal sex steroids. For example, using the delayed-type hypersensitivity response to *Candida albicans*, work from this laboratory has shown female responses are more robust than those of males (6), but ovariectomy increases rather than decreases the response (6). Moreover, estradiol replacement dampens the response (6). This is in agreement with studies from other laboratories, which likewise show a dampening of multiple types of responses by sex steroids in adults (7–14). Thus, although differential dampening by sex steroids in males and females remains a possible contributor to the greater female response, here we considered additional possibilities such as long-lived effects of prior exposure to gonadal hormones (organizational effects) and differences in the sex chromosomal complement.

Chromosomal differences can arise either from the presence of the Y chromosome in males or from gene dosage effects due to incomplete inactivation of the X chromosome in females (15, 16). To interrogate the potential contributory roles of organizational hormonal effects and chromosomal complement, we used gonadectomy and hormone manipulation in the “four core genotypes” (FCG) mouse model.

In this work, we considered the possibilities that sex differences might be present at birth as a result of chromosomal complement or manifest shortly thereafter as a result of the differential perinatal endocrine environment in the two sexes. In regard to the latter, while one normally associates sex steroids with puberty, there is an additional perinatal surge of testicular testosterone that occurs in males, peaking around the day of birth and falling within the adult range for concentration (17). There is no equivalent production of testosterone or estradiol in females.

Because of the immaturity of perinatal immune responses, our examination of sex differences was by necessity confined to numbers of cells and influences on markers of proliferation and apoptosis. Using these measures, we found no evidence of an effect of an XX *versus* XY chromosomal complement. However, perinatal hormonal manipulation uncovered heretofore unrecognized roles for sex steroids in thymocyte proliferation, thymic egress and splenic T cell seeding. In addition, we found evidence of a sex biasing effect specific to developing male CD8+ cells that was neither dependent on chromosomal complement nor the perinatal surge of testosterone.

## Methods

### Animals

FCG mice were obtained from the Jackson Laboratory (Bar Harbor, ME) on a C57BL/6J background and bred and housed under specific pathogen-free conditions, with controlled temperature, humidity and 12h light/dark cycles. In the FCG mouse model, the testis-determining gene, *Sry*, has been “moved” from the Y chromosome to an autosome, allowing the Y**^-^** chromosome and *Sry* to segregate independently. Mating XY^-^ *(Sry+)* gonadal males with XX gonadal females yields four genotypes (18): XX females, XY^-^ females that have equivalent levels of estradiol as adults (19), and XX*Sry+* males and XY^-^ *Sry+* males that have equivalent levels of testosterone both as adults (20, 21) and perinatally (22). These genotypes are henceforth abbreviated XXF, XYF, XXM and XYM, respectively. By comparing genotypes with the same gonads but different chromosomal complement, one can determine whether gonadal hormones or gene expression from XX or XY sex chromosomes causes a sex difference.

Anogenital distance was measured with calipers from the middle of the anus to the base of the penis. Measurement occurred without knowledge of the XXM *versus* XYM genotype. For genotyping, DNA was extracted from tail clips using the Qiagen (Valencia, CA) DNEasy kit per manufacturer instructions. Classical PCR was performed using the genotyping protocol recommended for FCG mice by Jackson laboratories (21). We define “male” as a mouse with testes, and “female” as a mouse with ovaries.

Gonadectomy of adults was performed at 10 weeks of age under isoflurane anesthesia. Tissues were collected 4 weeks after gonadectomy to reduce any effects of the surgery or lingering effects of gonadal hormones on immune parameters. All drug/hormone treatments used sesame oil/ethanol as control and diluent and were delivered subcutaneously every day after creating an emulsion by multiple passages through a 28-gauge needle. Flutamide was used at 12.5mg/Kg in neonates and 35mg/Kg in pregnant females (23), the latter delivered for 4 days before parturition, letrozole at 20 μg/Kg (24), testosterone at 5mg/Kg (23) and estradiol at 25μg/Kg (25) in neonates, all from Sigma-Aldrich, St Louis, MO. Treatment with SiRNA was at 1, 3 and 5 days after birth. Sry SiRNA or Control SiRNA (Thermo Fisher Scientific, Carlsbad, CA) was diluted in sterile Dulbecco’s phosphate buffered saline, complexed with invivofectamine 2.0 (Thermo Fisher Scientific) and dialyzed prior to administration, as recommended by the company. The number of animals contributing to a datum point is given in the figure legends.

### Tissue/Cell Collection

At the various time points, mouse pups were sacrificed, blood was collected into heparinized tubes, and the thymus and spleen harvested and placed in RPMI 1640. For flow cytometry, each blood sample was processed separately, but for assay of testosterone, plasma from sets of 10-14 pups from several litters was pooled, with replicates from different litters. To obtain individual cells, lymphoid organs were pressed through a 70 µM cell strainer using the soft plunger from a disposable 1 mL syringe. The number of cells obtained from neonatal lymphoid organs was limited, thereby restricting the variables that could be tested on cells from a single animal while making sure there were enough event counts during flow cytometry. Therefore, all experiments analyzing additional variables included one previous stain to ensure comparability among litters over time. Animal replicate numbers were also generally high when endocrinologically manipulating neonates in order to strengthen the data produced. When processed for epithelial/stromal microarray analysis, thymocyte removal and remnant tissue washing was repeated 5 times in the presence of RNase OUT (Invitrogen, Carlsbad, CA). The tissue remaining after removal of all thymocytes was snap frozen in liquid nitrogen and stored at -80°C until RNA extraction for microarray analysis.

### Microarray Analysis

RNA was extracted from the epithelium/stroma using a Qiagen RNeasy mini for Microarray Analysis kit as per the manufacturer’s instructions. An on-column DNAse digestion step was performed. Target preparation/processing was performed by the DNA & Protein MicroArray Facility, University of California, Irvine. All RNA samples were quality assessed prior to beginning target preparation/processing steps using an RNA 6000 Nano LabChip and evaluation on an Agilent Bioanalyzer 2100 (Agilent Technologies, Palo Alto, CA). Analysis used probe sets present on an Affymetrix mouse GeneChip 1.0 ST array (Santa Clara, CA). Arrays were scanned using the GeneChip Scanner 3000 7G and GeneChip Operating Software v. 1.4 to produce.CEL intensity files. Using the statistical environment R (R Core Team, 2014), expression values were extracted from.CEL files and normalized using RMA algorithm (R package “simpleaffy”). After the quantile normalization, two-way ANOVA was performed to identify the differentially expressed genes, with factors of gonadal sex (male vs. female) and sex chromosome complement (XX vs. XY). Benjamini-Hockberg (26) False Discovery Rate calculations were applied. Data have been uploaded to GEO: GSE 135071.

### RT-QPCR

RNA was quantified by nanodrop and equivalent amounts of RNA were reverse transcribed using Oligo(dT)_12–18_ Primer (18418-012 Invitrogen, Carlsbad, CA), M-MLV Reverse Transcriptase (28025-013 Invitrogen), RNase OUT, and 10 mM DNTP mix (invitrogen) for 1 hour at 37°C. Gene expression was analyzed by Quantitative PCR using 2x SYBR Green (Biorad, Hercules CA) and primers for GAPDH, Glcci, Wisp3, aromatase (CYP19A1), sphingosine kinase (Sphk), and sphingosine-1-phosphate lyase (Sgpl1). Primer sequences were: GAPDH (forward)-TGCACCACCAACTGCTTAG, (reverse)- GGATGCAGGGATGATGTTC, Glcci1 (forward)-ACACCTAGTTGCTGGGCAGA (reverse)-CTGCGTTGTAGCTGTTGCCT, Wisp3 (forward)-CGCTTCTCCATCTCTCCATCCT (reverse)- GTCTTGTGGTGCACTGCCCT. CYP19A1 (forward)- CTCAAGGGCGAGATGATAAGGT (reverse)- TCCTGTCACTTGGAAGGGTG, Sphk (forward)- ATGTGGTGGTGTTGTGTTTTGT (reverse)- CATGGTTCTTCCGTTCGGTG, Sgpl1 (forward)- GCCTCTGCGGGGAAAGAAG (reverse)- CAGGACACTCCACGCAATGA. Quantitative PCR was performed with initialization at 95°C for 5 minutes, followed by 40 cycles of denaturation at 95°C 10 seconds and annealing 30 seconds. After each run, a melt curve was run to assess appropriately amplified products.

### Flow Cytometry

Reagents and antibodies were purchased from eBioscience (San Diego, CA), BD Biosciences (San Jose, CA), R&D Systems (Minneapolis MN), Abcam (Cambridge, MA), Alamone labs (Jerusalem, Israel) or Santa Cruz Biotechnology (Santa Cruz, CA). Antibodies included anti-CD4 (RM4-5), anti-CD8 (53-6.7), anti-Ki-67 (SoIA 15), anti-CD90.1 (HIS 51), anti-CD44 (IM7), anti-c-Kit (2B8), anti-CD25 (PC61.5), anti-Sca-1 (D7), anti-Bax (B-9), anti-Qa2 (69H1-9-9), anti-S1PR1 (713412), anti-SPHK1 (ab95400), anti-S1PR3 (ASR-013-AG), mouse hematopoietic lineage cocktail (17A2, RA3-6B2, M1/70, TER-119 and RB6-8C5) and mouse IgG2a, IgG2b and rat IgG2b isotype controls, as appropriate. Anti-androgen receptor (AR antibody N-20) and anti-glucocorticoid receptor (GR antibody H-300) were rabbit polyclonal IgGs and controls included use of a non-specific rabbit polyclonal and second antibody alone. Fc receptors were blocked using purified rat anti-mouse CD16/CD312 Ab (2.4G2) (0.5 µg/100 µL). Intracellular staining was performed after cells were stained for surface antigens, fixed and permeabilized (BD Biosciences). Flow cytometry acquired intact cells, which were further gated on the basis of forward *versus* side scatter to identify lymphocytes and then analyzed after staining with conjugated Abs. An example is provided as part of the **Supplementary Figures**. This example shows forward *versus* side scatter, followed by analysis of cells positive for CD4 and/or CD8 and then, in this case, expression of sphingosine 1 phosphate receptors 1 and/or 3. For AR and GR staining, fluorochrome-conjugated mouse anti-rabbit IgGs were used as secondary antibodies. For compensation controls, fluorochrome conjugates along with non-stained controls were used to stain BD Biosciences CompBeads to set the equipment. In each Ab analysis, appropriate isotype controls were used to define positivity and gate settings.

### Statistical Analysis

Analysis of microarray data is described above. Excel statistical software was otherwise used for two-way ANOVA [factors of sex chromosome complement (XX vs. XY), and *Sry* (present vs. absent)] to assess group differences. Subsequently, pairwise comparisons were made using Tukey’s test for multiple comparisons or a simple Student *t* test for single comparisons. A p-value < 0.05 was considered significant. Effect sizes were computed by subtraction of the experimental mean from the control mean divided by the control standard deviation. Because our goal was to understand development of relative immune competency, which is impacted by numbers and relative numbers of cells, data are frequently presented as numbers of cells within a compartment or this plus percentages. The presentation of numbers of cells increases the errors because of variability in cell recovery from the organs and errors due to variation in animal size, particularly among neonates. However, presentation of absolute numbers eliminates confounding aspects of interpretation in percentage changes. i.e. a percentage may increase because the numerator was increased or the denominator was decreased. Litters were normalized to 5 ± 1 and runts were eliminated from analyses. Flow cytometry was conducted before genotyping and by a different individual to ensure blinded analysis. The number of animals contributing to a datum point in the various experimental groups is provided in the figure legends. Although over the longer term, one would expect an even distribution of genotypes, rarely was this observed in a given litter. All data are derived from pups from multiple litters, obtained and analyzed on more than one occasion.

## Results

### Sex Differences in Gonadectomized Adult FCG Mice

**Figure 1:**
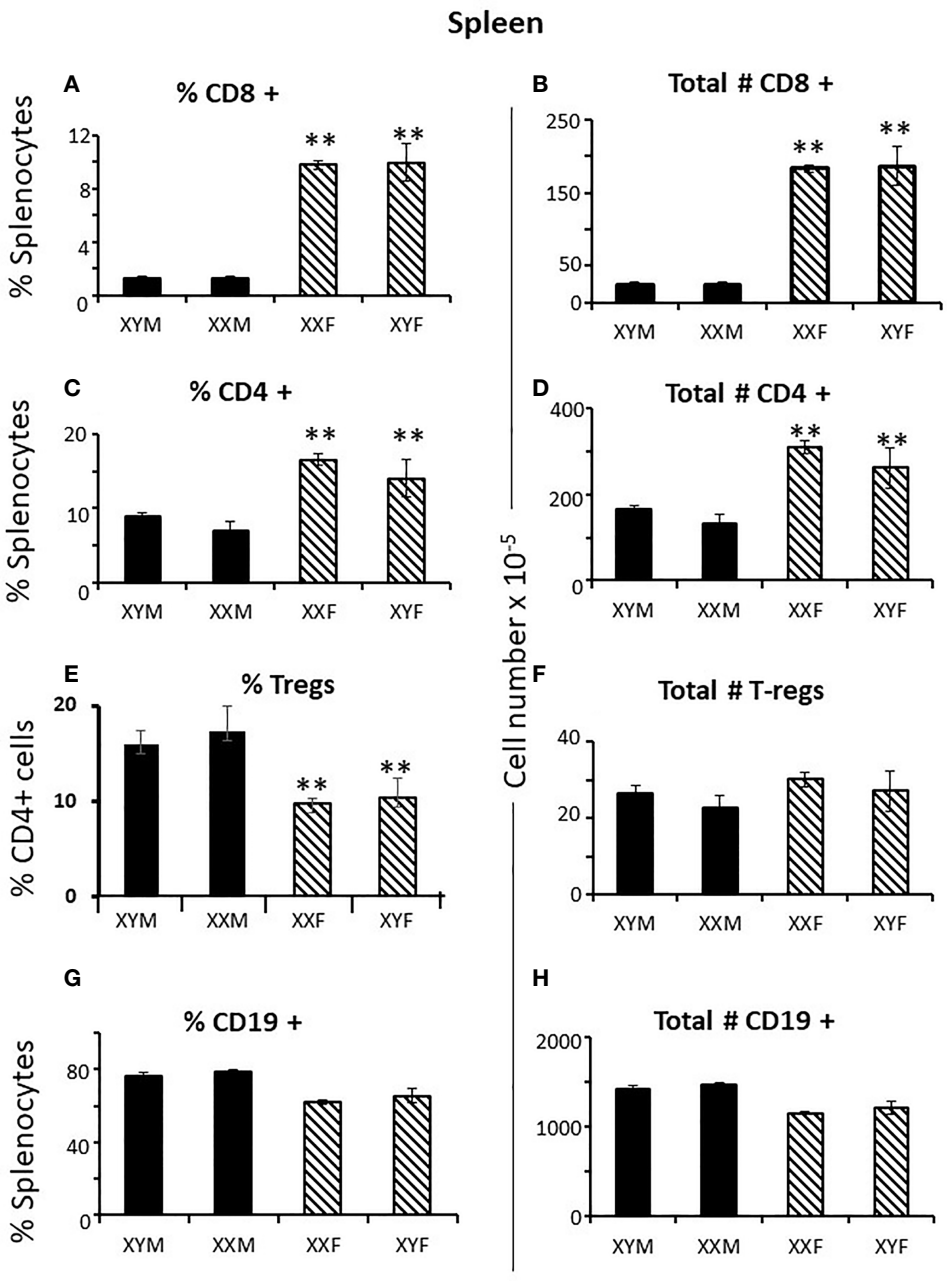
Cellular content (percentages and numbers) of adult FCG spleens 4 weeks after gonadectomy. Data are presented as a percentage of gated lymphocytes present in the spleen except for Tregs (CD4+CD25+Foxp3+), which are presented as a percentage of CD4 single positive cells. Data are the mean ± SD and are derived from 3-5 animals per genotype. **, different from both male genotypes, p<0.01.

To establish measures of sex differences in the adult immune system that could be applied to neonatal mice having immature and inexperienced immune systems, we analyzed numbers of CD4+ and CD8+ T cells in the spleen and thymus, and numbers of CD19+ B cells and T regulatory cells (Treg) in the spleens of gonadectomized adults. CD4+ T cells are helper cells that stimulate other immune cells, including CD8+ T cells and B lymphocytes. CD8+ T cells are cytotoxic cells responsible for killing cells containing intracellular pathogens, such as viruses and some bacteria, and cancer cells expressing abnormal proteins. B cells will differentiate into antibody-producing cells, and Treg cells (formerly known as T suppressor cells) moderate immune responses, maintain self-tolerance and prevent autoimmune diseases. Adult FCG mice were gonadectomized at 10 weeks of age and analyzed 4 weeks later in order that any effects of the surgery on immune cell numbers or lingering, reversible effects of gonadal hormones would have dissipated. Two-way ANOVA showed a significant main effect of sex, but no effect of sex chromosome complement (XX vs. XY), and no significant interaction between sex and sex chromosomal complement. There were very different numbers of CD8+ and CD4+ T cells in the spleens of females and males; the XYF and XXF had 7-fold the number of CD8+cells and twice as many CD4+ cells when compared to the XYM and XXM (**Figures 1A–D**). Thus, even 4 weeks after removal of adult gonadal hormones, a difference based on gonadal sex was evident in the spleen. Using this measure, there was no difference based on chromosomal complement since both male numbers (XXM and XYM) and both female numbers (XXF and XYF) were the same.

**Figure 2:**
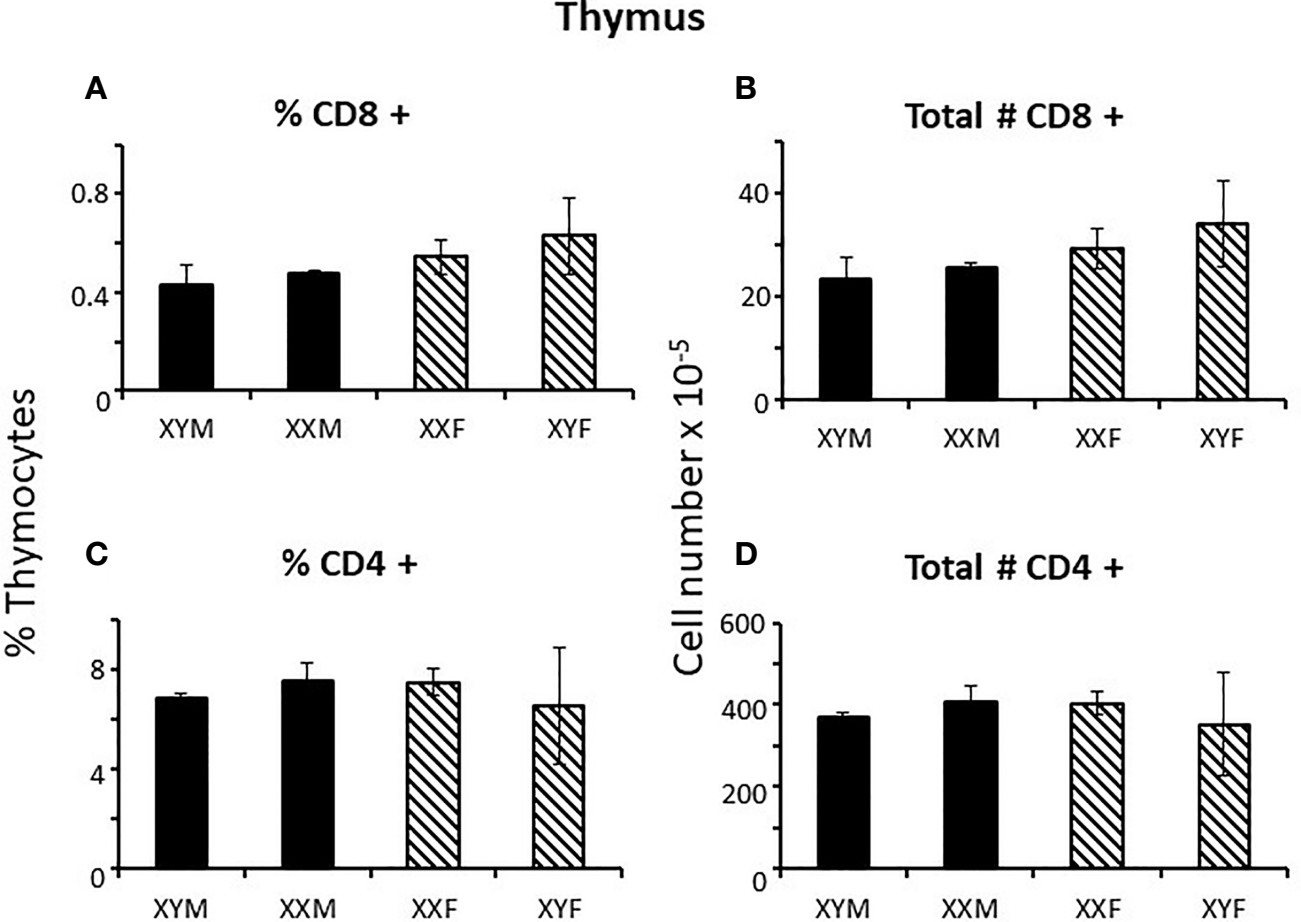
Cellular content (percentages and numbers) of adult FCG thymi 4 weeks after gonadectomy. Data are presented as a percentage of gated thymocytes (mean ± SD) and are derived from 3-5 animals per genotype.

Immune responses are dependent on both the overall number of effector cells and the degree to which effector cells are constrained by Tregs (27); no sex difference was observed for Treg numbers in the spleen. However, a greater number of potential effector T cells in females and the same number of Tregs results in a lower percentage of Tregs in females (**Figures 1E, F**) which could theoretically manifest as more profound immune responses, depending on their specificities. For B cells, there was no overt effect of sex or sex chromosomal complement (**Figures 1G, H**). Sex differences in T cell numbers or percentages were not reflected in the thymus (**Figures 2A–D**) where there was also no apparent effect of chromosomal complement or their interaction on cell number.

### Sex Differences in Neonatal FCG Mice

**Figure 3:**
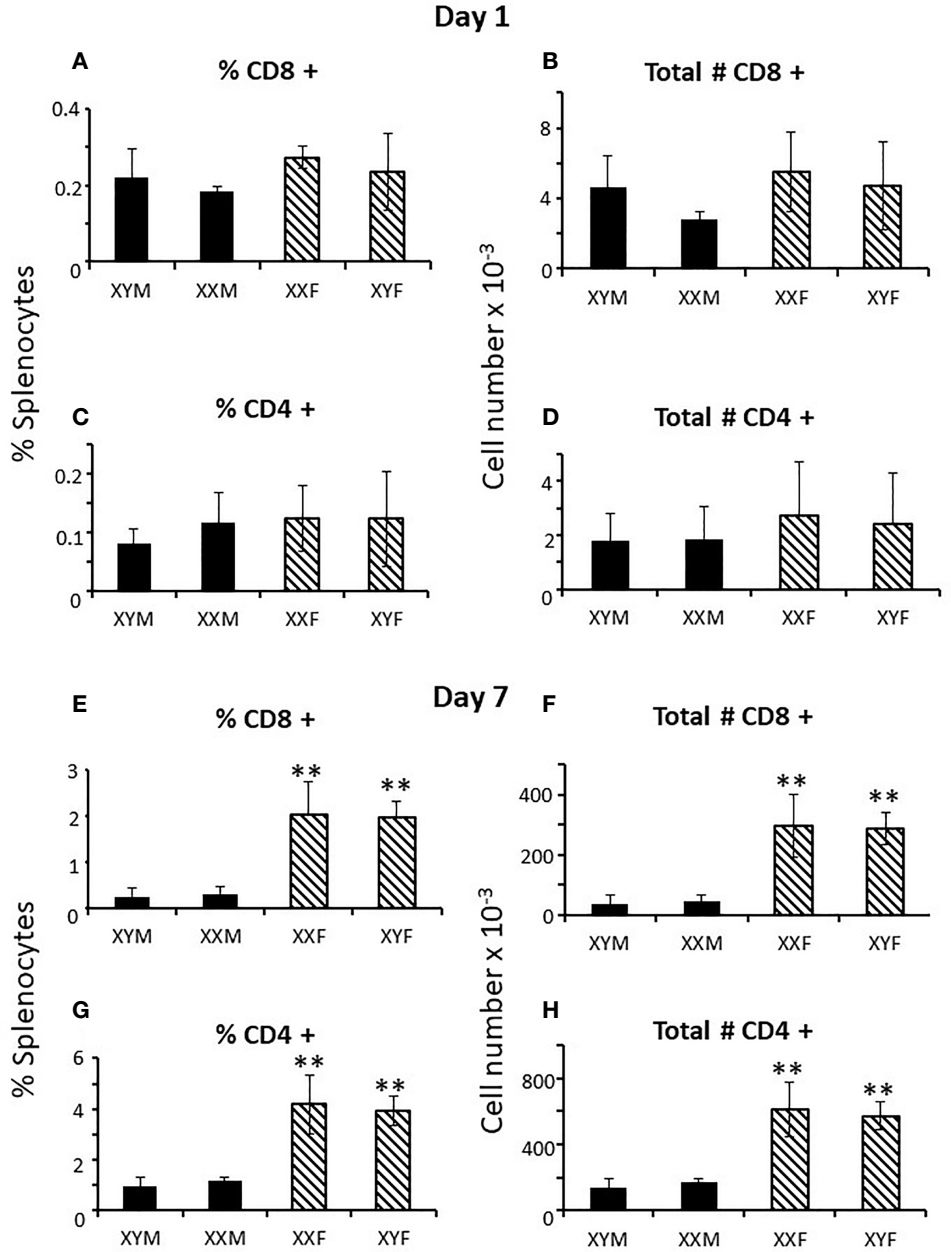
Mature T cell content (percentages and numbers) of the spleens of pups at 1 and 7 days of age. Data are presented as a percentage of gated lymphocytes. The means ± SD are derived from 17 XYM, 14 XXM, 15XXF, and 16 XYF animals at day 1, and 13 XYM, 14 XXM, 22 XXF, and 13 XYF animals at day 7. **, different from both male genotypes, p < 0.01.

To determine when sex differences in splenic T cell number became apparent, FCG neonates were examined at 1 and 7 days postpartum. At day 1, there was no evidence of CD8+ or CD4+ sex differences in the spleens, whether plotted as all males *versus* all females (not shown) or by genotype (**Figures 3A–D**). However, by 7 days of age, full splenic sex differences were evident, and, if anything, for CD4+ cells was greater than in the adult (**Figures 3E–H**). Thus, sex differences became apparent during the first week of life. This result does not preclude a prenatal effect of sex-biasing factors that may have taken some time to become evident. Nevertheless, it does establish the time period during which the sex differences become physiologically relevant to immunity. As was true for the adult, no sex difference was evident for numbers of cells in the thymus or for Tregs or B cells in the spleen at 7 days (**Supplementary Figure 1**).

In newborn mice, the spleen serves as an hematopoietic organ since mice are born with a cartilaginous skeleton and essentially no bone marrow cavity is initially available for this function (28). Profound sex differences in T, but not B, cells suggested the effect of gonadal sex was not on an early hematopoietic precursor giving rise to both B and T cells. As anticipated, analysis of splenocytes for the common T and B cell hematopoietic progenitor (Lin-thy1loCD44+Sca+ckit+) showed no difference in number between males and females when assessed for individual genotypes (not shown) or when combining both male and both female genotypes (**Table 1A**).

Table 1Cell values 7 days postpartum comparing both male to both female genotypes.

**Table:** 

**A: No treatment**						
	Spleen	Spleen	Spleen	Blood	Blood	Spleen
	Number hematopoietic progenitors Lin-thy1loCD44+Sca+ckit+ (x 10^-3^)	Percent CD4+ also Ki67+	Percent CD8+ also Ki67+	Percent CD4+Qa2lo	Percent CD8+Qa2lo	Number thymic progenitors CD4-CD8-thy1loCD25-CD44+ckit+ (x 10^-5^)
Male	14.7 ± 6.8	46.6 ± 7.1	75.9 ± 5.2	57.2 ± 18.6	42.4 ± 20.7	1.86 ± 0.8
Female	18.0 ± 13.5	52 ± 7.4	75.5 ± 6.3	68.7 ± 16.5	43.9 ± 20.3	1.42 ± 0.9
	**Thymus**	**Thymus**	**Thymus**	No male-female differences were significant		
	Number thymic progenitors CD4-CD8-thy1loCD25-CD44+ckit+(x 10^-3^)	Percent total thymocytes Ki67+	Percent total thymocytes Bax+			
Male	3.3 ± 1.9	92.4 ± 3.4	51 ± 29.7			
Female	3.7 ± 2.4	91.8 ± 1.3	68.2 ± 37.8

**Table:** 

**B. After treatment with flutamide or letrozole between days 1 and 7.**				
	Percent double negative thymocytes also Ki67+	Percent double positive thymocytes also Ki67+	Percent CD4+ thymocytes also Ki67+	Percent CD8+ thymocytes also Ki67+
Male	64.5 ± 5.7	92.7 ± 4.3	92.4 ± 3.0	97.0 ± 1.8
Male + Flu	67.4 ± 1.8	85.1 ± 2.8	83.9 ± 6.1	94.3 ± 2.7
Male + Let	65.6 ± 8.9	84.6 ± 5.2	84.7 ± 5.5	91.4 ± 6.2
Female	69.4 ± 4.7	91.5 ± 1.8	93.3 ± 3.8	96.6 ± 3.5
Female + Flu	68.0 ± 3.6	83.1 ± 3.0 *	80.4 ± 6.6 *	92.9 ± 2.3
Female + Let	61 ± 17.4	78.7 ± 17.5 *	80.6 ± 11.0 *	89.1 ± 10.6

All values mean ± SD. *, different from control, p<0.05.

T cell sex differences in the spleen at 7 days could have arisen by differential proliferation of T cells once in the spleen, differential T cell egress from the spleen or differential seeding from the thymus. Focusing first on proliferation and using Ki67 as a marker of proliferative capacity, no difference was observed in the percentage of CD8+ or CD4+ cells that were Ki67 positive between males and females when assessed in individual genotypes (not shown) or when combining both male and both female genotypes (**Table 1A**). i.e. there was no evidence of preferential proliferation of cells in the female spleen. Adding to general information about neonatal immunology, however, we did note a much higher percentage of CD8+ *versus* CD4+ cells was Ki67+ at 7 days of age irrespective of sex (75% *versus* 48%, respectively). This was an unexpected feature since neonates are widely considered to have poor cytotoxic T cell responses (29).

**Figure 4:**
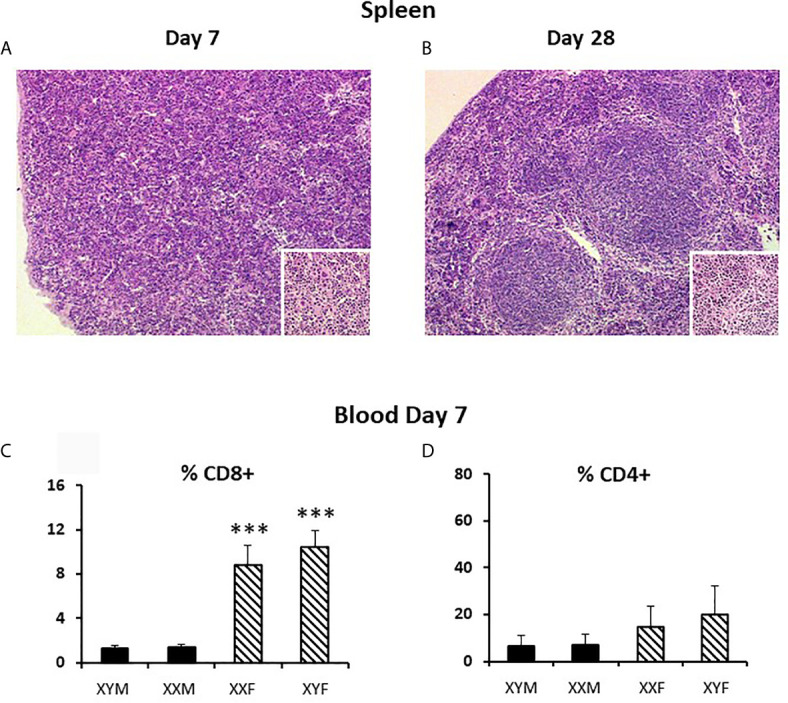
Pup spleen histology at 7 and 28 days and T cell content of the blood at 7 days. Spleens were fixed and embedded in JB4 (a water soluble, glycol methacrylate-based embedding medium) before sectioning (@ 2.5 μm) and staining with hematoxylin and eosin. Magnification, 40X and 100X for insets. Blood was collected into heparinized tubes and the red blood cells lysed prior to staining for flow cytometry. Data in c and d are presented as a percentage of gated lymphocytes. The means ± SD are derived from 13 XYM, 14 XXM, 22 XXF, and 13 XYF animals at day 7. ***, different from both male genotypes, p<0.001.

During this first week of life, splenic white pulp is not yet structured into T and B areas (compare **Figure 4A** with **B**), is actively accumulating cells, and contributes few cells to the blood (30). In the absence of sex differences in splenic T cell proliferation and with little contribution to the blood, T cell sex differences in the spleen seemed therefore most likely due to differential seeding from the thymus.

Differential seeding from the thymus to the spleen *via* the blood is supported by analysis of blood lymphocytes at 7 days, which showed the same degree of sex difference as the spleen for CD8+ cells (**Figure 4C**). The means for CD4+ cells also hinted at a similar situation, but the differences were not statistically significant (**Figure 4D**).

There are a variety of markers associated with post-thymic maturation, one of which is the expression of Qa2. As cells mature in the periphery, the expression of surface Qa2 moves from low to high (31). There were no main effects of sex or sex chromosomal complement, nor an interaction of these two in the percentage of cells expressing low levels of Qa2 between males and females for either CD8+ or CD4+ cells (**Table 1A**). So, once in the circulation, male and female cells mature at the same rate.

In the first week postpartum, the thymus is in high production mode to supply the blood, connective tissues, and secondary lymphoid organs. At 7 days, neither numbers of specific T cell progenitors (CD4-CD8-thy1loCD25-CD44+ckit+) in the spleen or thymus, nor percentages of Ki67+ or Bax+ thymocytes, were different between males and females (**Table 1A**). Unexpectedly, a large proportion of cells were positive for both Ki67 and Bax. However, concurrence of parameters used as markers of both proliferation and apoptosis has been previously reported for thymocytes in a study using DNA content as a measure of apoptosis and both Ki67 and proliferating cell nuclear antigen as proliferation markers (32).

**Figure 5:**
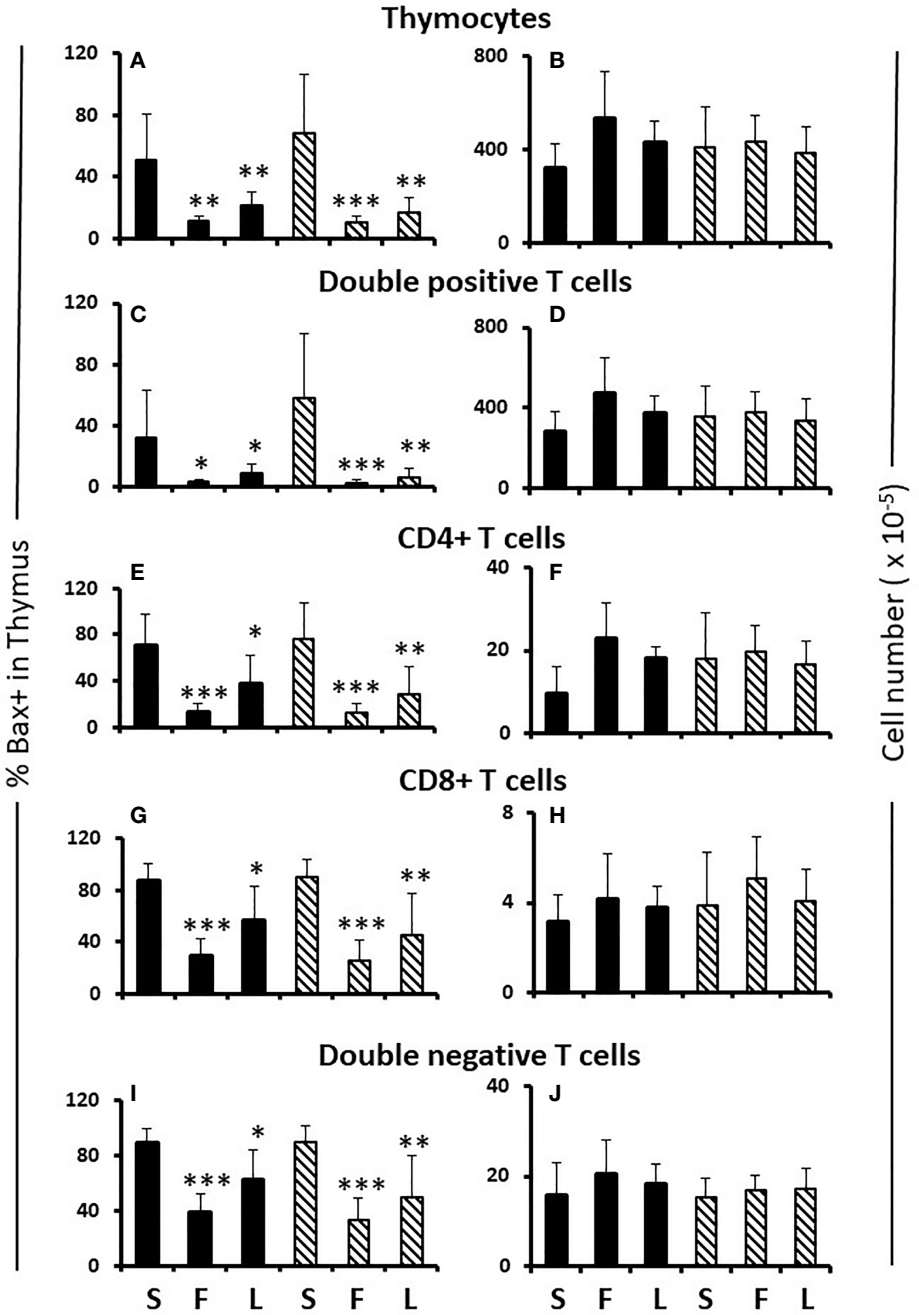
Effect of flutamide and letrozole on the percentage of cells expressing Bax and the number of cells in the thymus of pups. FCG Mouse pups were treated between days 1 and 7 postpartum before collection of thymi. Data are presented as a proportion of gated lymphocytes. The means ± SD are derived from 6 males and 8 females in the sesame oil control group, 7 males and 13 females in the flutamide group and 11 males and 7 females in the letrozole group. Males include both XYM and XXM (solid bars) and females include both XXF and XYF (cross-hatched bars). S, sesame oil control; F, flutamide; L, letrozole. Different from same sex control *p < 0.05; **p < 0.01; ***p < 0.001.

The results also show no significant difference in percentage of Ki67+ or Bax+ cells between males and females when analyzing different thymocyte developmental stages in control, sesame oil-treated animals either by individual genotype (not shown) or by gonadal sex (**Table 1B** and **Figures 5A, C, E, G, I** sesame oil control values). Cells progress from being CD4CD8 double negative through CD4CD8 double positive to either CD4 or CD8 single positive before release from the thymus. There was also no difference in numbers of cells between males and females at different developmental T cell stages (**Figures 5B, D, F, H, J** sesame oil control values). With no difference in numbers, the rates of proliferation, as indicated by Ki67, and apoptosis, as indicated by Bax positivity, appear to be the same for both sexes. However, if there were different rates of exit from the thymus, this conclusion would not be accurate, as discussed later. Because relative expression of Bax per cell might have produced differential susceptibility to apoptosis, this was also analyzed for each T cell developmental stage. Again, relative expression per cell was the same in males and females (**Supplementary Figure 2** sesame oil control values).

### Hormonal Effects on the FCG Neonatal Spleen

**Figure 6:**
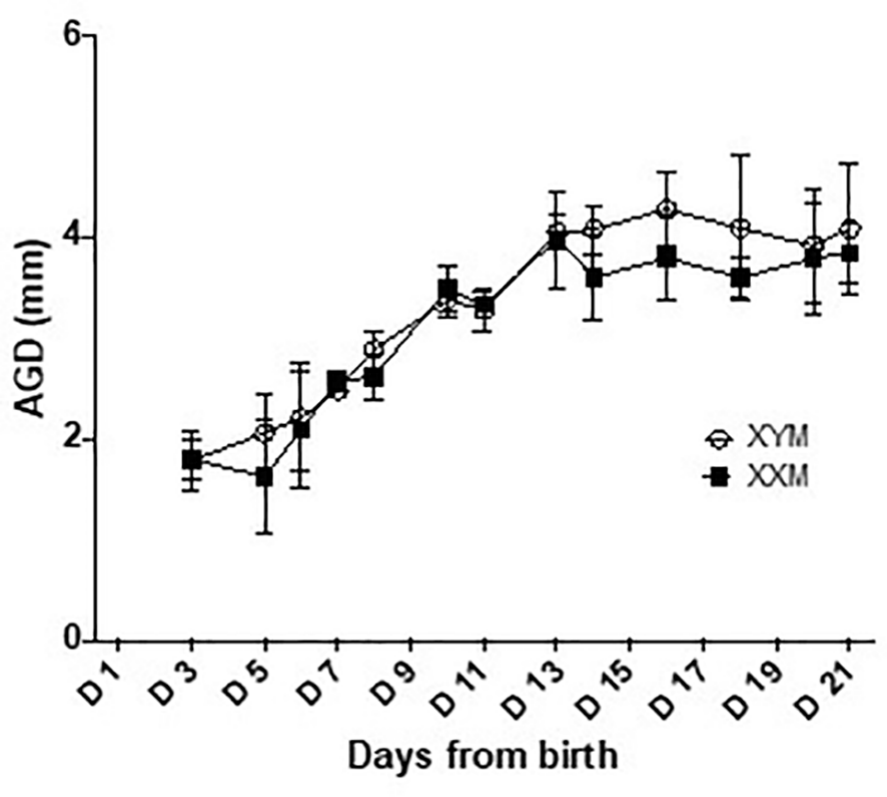
Anogenital distance development in FCG male pups. Anogenital distance was measured daily until weaning. Data are presented as the mean ± SD. No significant difference was found between XYM and XXM genotypes on any day analyzed by repeated measure 2-way ANOVA with post-hoc Sidak corrections for multiple comparisons. n = 4 XXM and 5 XYM pups.

In males, there is peripartum, testicular Leydig cell production of testosterone, peaking around the day of birth, and falling within the adult range for concentration (17). There is no equivalent production of testosterone or estradiol in females. Limited sampling confirmed a testosterone surge in male FCG mice and equivalent levels in both adult male genotypes (**Supplementary Table 1**). In addition, previous work by Itoh et al. (22) demonstrated no difference in the anogenital distance of FCG mice, suggesting that perinatal exposure to testosterone is approximately equivalent between the XXM and XYM genotypes. In the present study, we have confirmed the result of Itoh et al. (22) that XX and XY FCG males have indistinguishable rates of increase in anogenital distance (**Figure 6**). These data confirm the likelihood that both FCG male genotypes are exposed to similar perinatal levels of testosterone.

**Figure 7:**
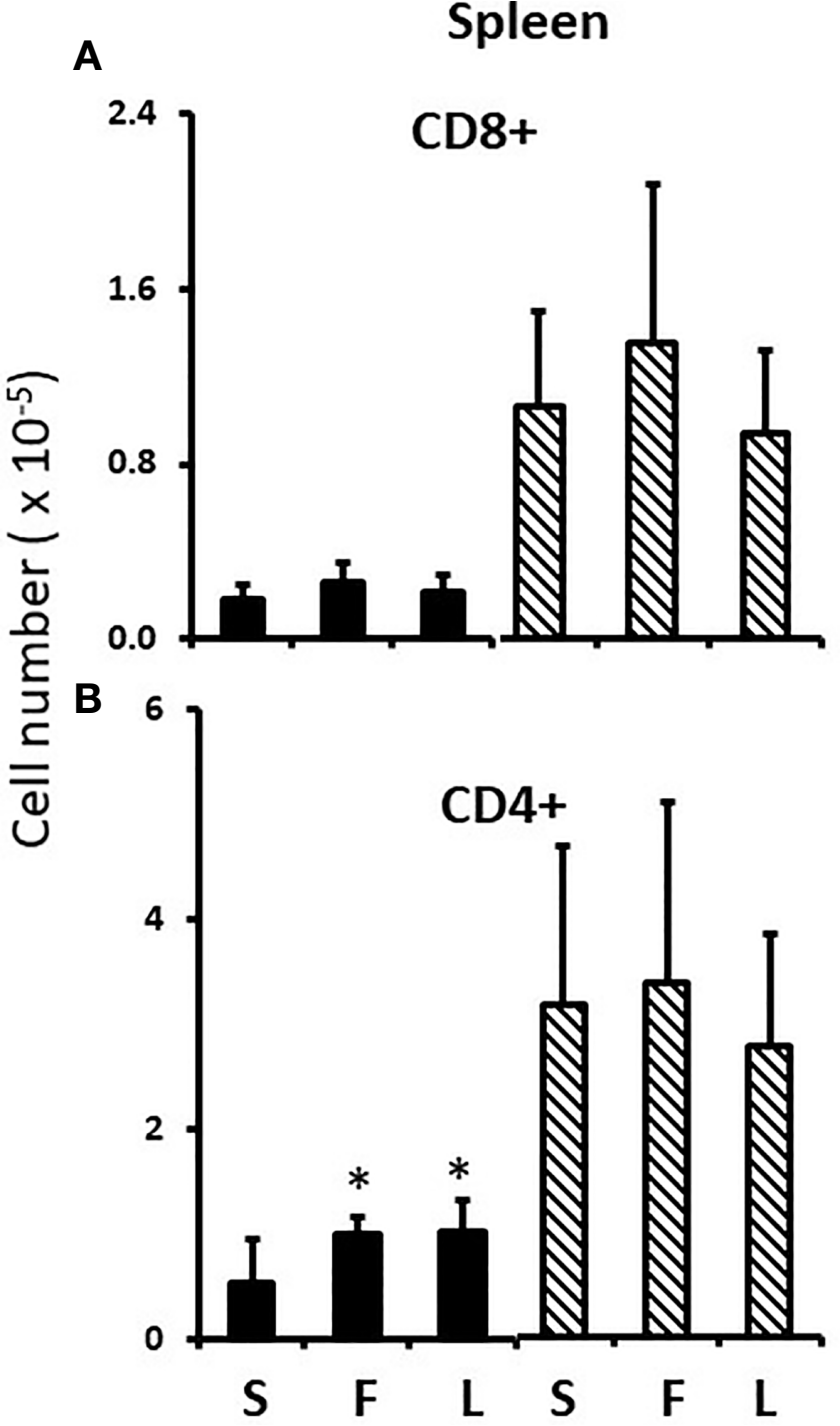
Effect of flutamide and letrozole on numbers of cells in the pup spleen. FCG Mouse pups were treated between days 1 and 7 postpartum before collection of spleens. Data are derived from gated lymphocytes, presented as the mean ± SD, and are derived from the same animals as for **Figure 5**. S, sesame oil control; F, flutamide; L, letrozole. Males include both XYM and XXM (solid bars) and females include both XXF and XYF (cross-hatched bars). Different from same sex control *, p< 0.05, effect size 1.25.

To test the potential role of perinatal testosterone in the development of the splenic male-female sex differences, we employed the competitive antagonist, flutamide (33), with the expectation that flutamide would block male-typical development of the splenic profile and increase the number of T cells. Because perinatal testosterone is often aromatized to estradiol to accomplish its masculinizing effects (21), we also tested if the aromatase inhibitor, letrozole, blocked masculinization (34). Because of the potential to alter the dam’s endocrinology and thereby complicate analyses specific to events occurring in the pups, the drugs were given to the pups between days 1 and 7 postpartum. Since a proportion of the peripartum surge would already have taken place, we expected that the drugs might cause significant, but incomplete blockade of masculinization. Both flutamide and letrozole had a significantly de-masculinizing effect and approximately doubled the number of CD4+ T cells in the FCG male spleens, with no effect on the number of CD4+ cells in the FCG female spleens (**Figures 7A, B**). Thus, perinatal testosterone was responsible for at least a large part of the decreased numbers of CD4+ cells in the male spleen. However, there was no evidence that the same was true for CD8+ cells, even though the sex differences were greater for CD8+ cells; neither flutamide nor letrozole significantly increased CD8+ cells in the male spleen (**Figure 7A**). The lack of effect of the drugs on cell content of the female spleen also indicated specificity of the effect.

### Effects of Flutamide and Letrozole in the FCG Neonate Thymus

Flutamide and letrozole caused a small, but significant, reduction in Ki67 positivity of CD4CD8 double positive and CD4 single positive cells in the female thymus (**Table 1B**) and both drugs resulted in a major reduction in the percentage of Bax+ thymocytes at all developmental stages in both sexes (**Figures 5A, C, E, G, I**). This result implied that a degree of proliferation of some subsets and substantial promotion of Bax expression/apoptosis of all thymocytes is brought about by testosterone aromatized to estradiol. Furthermore and importantly, since there is no peripartum surge of testosterone in females, the source of the steroids being affected by flutamide and letrozole was not testicular, at least in females. However, even with the imbalance between the degree of effect on Ki67 and Bax positivity, there was no net effect on thymic cell number (**Figures 5B, D, F, H, J**).

To determine whether the thymic epithelium could be the source of the testosterone and estradiol affected by administration of flutamide and letrozole, microarray analysis of gene expression in day 7 thymic epithelium/stroma was performed. Thymic epithelium/stroma showed expression of all necessary enzymes required to produce testosterone and its aromatization to estradiol, and no difference in expression between FCG gonadal males and females (**Supplementary Table 2**) or any of the genotypes (individual genotype data not shown). Although mRNA expression cannot assess relative activity of these enzymes, these data are at least consistent with the observed equivalent effects of flutamide and letrozole within the thymus of both males and females and the necessity for aromatization of testosterone to estradiol to promote end effects.

In keeping with the flutamide and letrozole results on Bax expression, others have described a major effect of testicular testosterone to promote T cell apoptosis in the thymus, mediated through testosterone stimulation of local corticosteroid production (35). As shown in **Supplementary Table 2**, androgen receptors (AR) are expressed in the neonatal thymic epithelium/stroma, as is CYP11A1, the enzyme responsible for production of pregnenolone and rate limiting for corticosterone synthesis (36), as well as CYP11B1 that converts 11-deoxycorticosterone into corticosterone. However, there was no difference in levels of expression between males and females. Thymocyte apoptosis in response to corticosterone would require expression of glucocorticoid receptors (GR). Flow cytometric determination of the percentage of cells expressing, or the expression level per cell, at days 1 and 7 of these or androgen receptors (AR) showed no difference between males and females at any thymocyte developmental stage (**Supplementary Figures 3** and **4**). These data are again consistent with equivalent effects of flutamide and letrozole on Bax expression in males and females.

Results to this point, while suggesting a potentially important role for intra-thymic local testosterone/estradiol in the regulation of thymocyte number, have not explained sex differences in splenic T cell number nor the male specificity of the effect of flutamide and letrozole on the numbers of CD4+ cells in the spleen. There must therefore be an additional mechanism of action of these drugs in males.

### Effects of Hormone Treatment on the FCG Neonate Thymus

**Figure 8:**
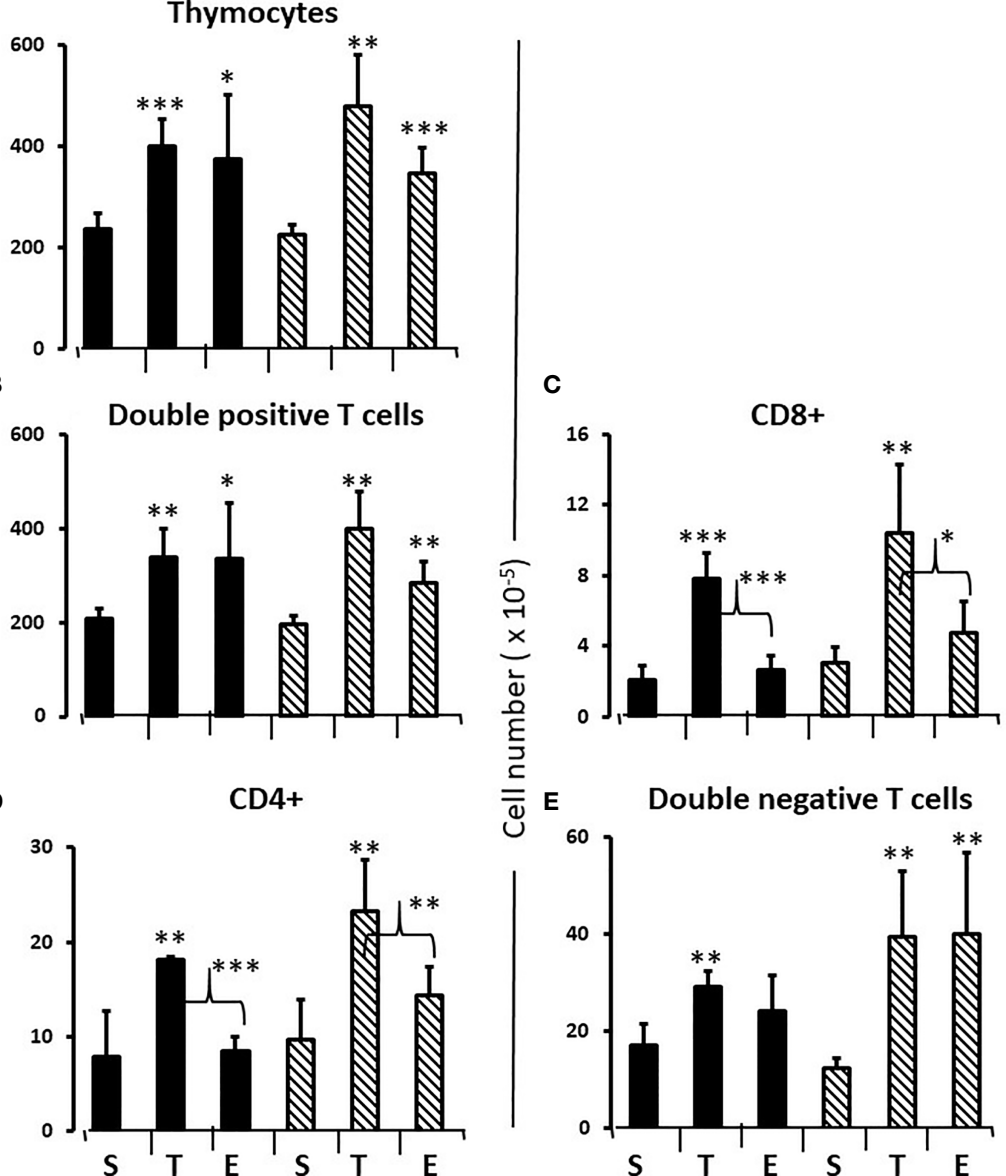
Effect of testosterone and estradiol on cell numbers in the thymus. FCG Mouse pups were treated between days 1 and 7 postpartum before collection of tissues. Data are derived from gated lymphocytes, presented as the mean ± SD, and are derived from 6 males and 5 females in the control group, 3 males and 3 females in the testosterone group and 5 males and 7 females in the estradiol group. S, sesame oil control; T, testosterone; E, estradiol. Males include both XYM and XXM (solid bars) and females include both XXF and XYF (cross-hatched bars). Brackets show difference between included bars, with the asterisk designating the degree of significance for these and those compared to same sex control *p < 0.05; **p < 0.01; ***p < 0.001.

To consolidate developing hypotheses and determine what additional effect testosterone had in males, FCG pups of both sexes were treated with testosterone or estradiol between days 1 and 7. Contrary to what one might expect given that the greater effect of flutamide and letrozole was to decrease Bax expression, both testosterone and estradiol increased total thymocyte, double positive and double negative cell number by about 60% (**Figures 8A, B, E**). For single positive cells, only testosterone increased cell number (both CD8+ and CD4+). (**Figures 8C, D**). In other words, there was also a testosterone-specific effect on cells mature enough to leave the thymus.

**Figure 9:**
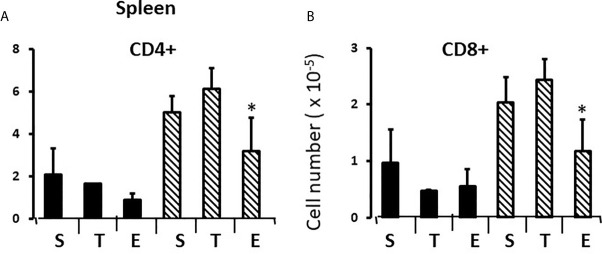
Effect of testosterone and estradiol on cell numbers in the spleen. FCG Mouse pups were treated between days 1 and 7 postpartum before collection of tissues. Data are derived from gated lymphocytes, presented as the mean ± SD, and are derived from 6 males and 5 females in the control group, 3 males and 3 females in the testosterone group and 5 males and 7 females in the estradiol group. S, sesame oil control; T, testosterone; E, estradiol. Males include both XYM and XXM (solid bars) and females include both XXF and XYF (cross-hatched bars). Different from same sex control *p < 0.05, effect size 2.3 and 2.0 for CD4+ and CD8+ cells, respectively.

**Figure 10:**
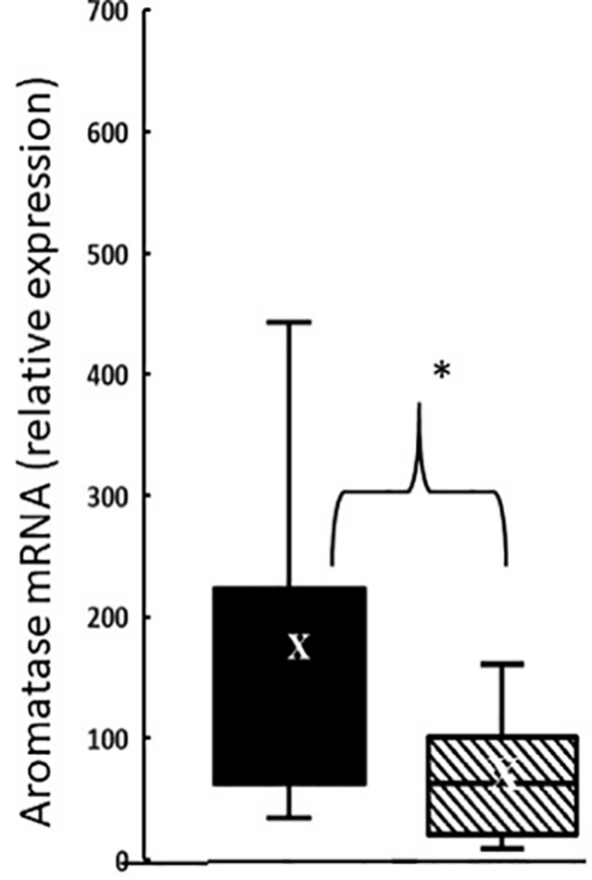
Aromatase expression at postnatal day 7 in splenic epithelium/stroma from FCG gonadal males *versus* gonadal females. Expression is normalized to GAPDH. Data are presented as a box and whisker plot and X denotes the mean. N = 13 male (both XYM and XXM) pups (solid bars); n = 11 female (both XXF and XYF) pups (cross-hatched bars). Males different from females, *p < 0.05, effect size 1.12.

In the thymus, the effects of treatment with testosterone and estradiol were very similar in males and females. However, under normal circumstances, only the male would experience a surge of testicular testosterone that would result in an increase in the number of single positive cells in the thymus. An increase in the number of single positive cells could be the result of increased production/maturation, or more likely given both the reduced number in the spleen and the difference from estradiol, from inhibition of final maturation/release. However, if the only difference between males and females contributing to sexual dimorphism in the spleen were inhibition of release of single positive cells from the thymus by testosterone in males, then we should have seen a reduction in single positive cells in the spleen of both males and females with testosterone treatment and we did not (**Figures 9A, B**). Although the trend was towards a reduction with additional testosterone administration in males, this was not duplicated in females. Furthermore, the trend in the male spleen was replicated by estradiol, which did not inhibit release from the thymus. In other words, there is another sex-specific effect at the level of the spleen independent of effects on the thymus that suggested the ability of the male, but not female spleen to aromatize testosterone. To determine whether there might be a differential ability of splenic stroma to aromatize testosterone to estradiol, expression of aromatase mRNA in splenic epithelium/stroma was examined by qPCR. **Figure 10** shows that epithelium/stroma from FCG males (XY and XX) expresses CYP19A1 at about two to three times the level of FCG females (XY and XX). Thus, making the assumption of relative enzyme activity on the basis of relative mRNA expression, spleens from FCG gonadal males do have increased potential for aromatization of testosterone to estradiol.

### CD8+ Cells

**Figure 11:**
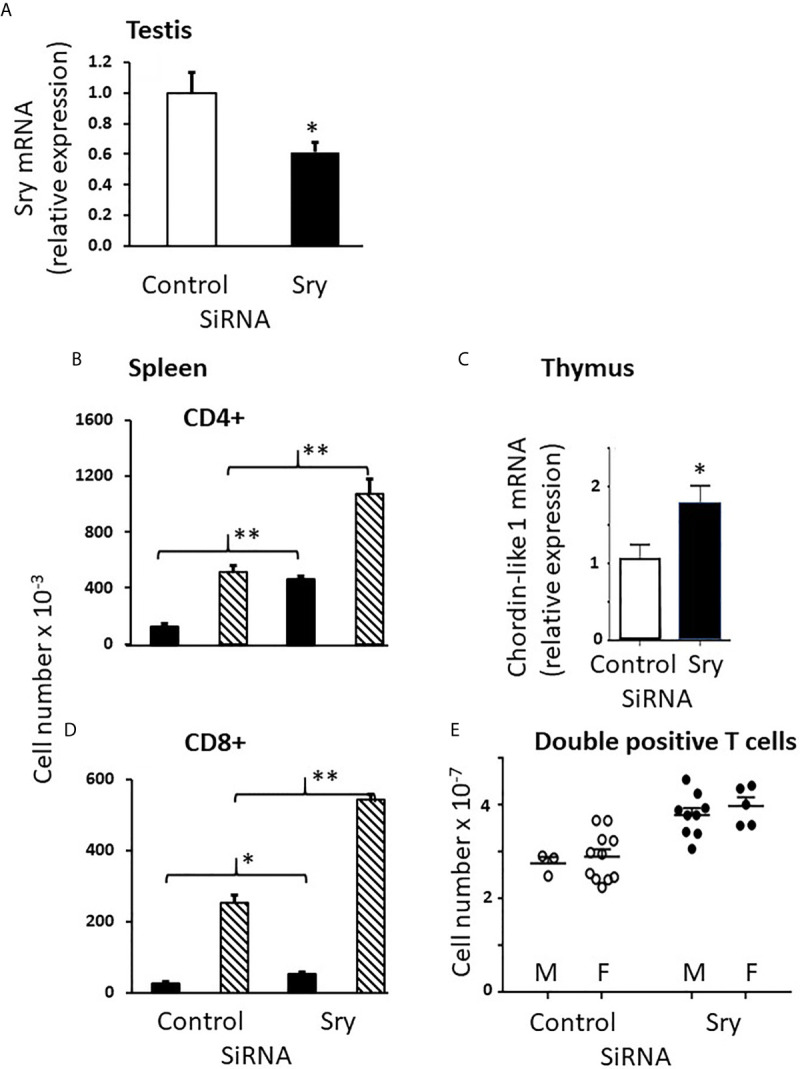
Use of the SiRNA for Sry had similar effects in male and female FCG pups. Knockdown of Sry within the male testes was confirmed **(A)**, but both male and female animals showed an increase in both CD4+ and CD8+ cells in the spleen as a consequence of treatment **(B, C)**. Use of the Sry SiRNA raised expression of chordin-like 1 **(D)** and increased the number of double positive cells in the thymus of treated animals **(E)**. Mouse pups were treated with the Sry or control SiRNA between days 1 and 7 postpartum before collection of tissues. Data are presented as the mean ± SD and are derived from 3 males and 11 females for control SiRNA treatment groups and 9 males and 5 females for Sry SiRNA treatment groups. Males (solid bars in **B**, **C**); Females (cross-hatched bars in **B**, **C**). Different from control SiRNA *p < 0.05; **p < 0.01.

Splenic sexual dimorphism is greatest for CD8+ cells. We therefore anticipated being able to see larger responses to experimental manipulation for these cells *versus* CD4+ cells, but that was not the case. Other than testicular testosterone, the only entity common to both male genotypes and not present in either female genotype was the *Sry* gene. We therefore used a commercially-available SiRNA to knock down Sry expression postnatally. The ability of this SiRNA to knock down Sry expression in the testes is shown in **Figure 11A**. Knockdown of about 40% compared to control SiRNA was achieved. If we had confined our analysis to males, the results would have supported a very important role for Sry expression in the development of immune sex differences since knockdown doubled the number of splenic CD4+ and CD8+ cells in males, largely feminizing the CD4+ profile and partially feminizing the CD8+ profile in the spleen (**Figures 11B, C**). Unfortunately, however, the same effect was observed in females (**Figures 11B, C**), indicating that the action of the SiRNA was not specific to Sry. Nevertheless, we felt it important to include the results here because a) it is an obvious experiment to perform and its absence would have been notable and b) it was important to draw attention to the lack of specificity of an SiRNA marketed as specific. Unfortunately, there is no viable, alternative SiRNA. After *in silico* analysis of potential non-specific effects of the SiRNA, we examined the effect of the “Sry” SiRNA on expression of Chordin-like 1 and found it was increased (**Figure 11D**). Chordin-like 1 is an antagonist of bone morphogenic protein-4, which in turn inhibits the progression of double negative to double positive thymocytes (37). Therefore, an increase in the antagonist of an inhibitor of T cell maturation would be expected to increase thymic output of T cells. This seems a likely explanation for the non-specific effect of the SiRNA. Supporting this explanation, **Figure 11E** shows an increase in double positive cells in the thymi of both FCG males and females after treatment with the “Sry” SiRNA.

Examination of the numbers of CD8+ cells in the spleens shows about equal numbers of CD8+ cells in FCG males and females at day 1 (**Figure 3B**), an ~ 10 fold increase from day 1 to day 7 in male genotypes, and an ~ 100 fold increase in female genotypes (**Figure 3F**), suggesting that the effect of gonadal sex on CD8+ cell production may already have been in place on day 1. To be sure that this was not an early effect of the perinatal testicular testosterone surge, pregnant dams were treated with flutamide for 4 days before parturition and splenic CD8+ cell numbers were examined on postnatal day 1. Flutamide had no effect (data not shown).

**Figure 12:**
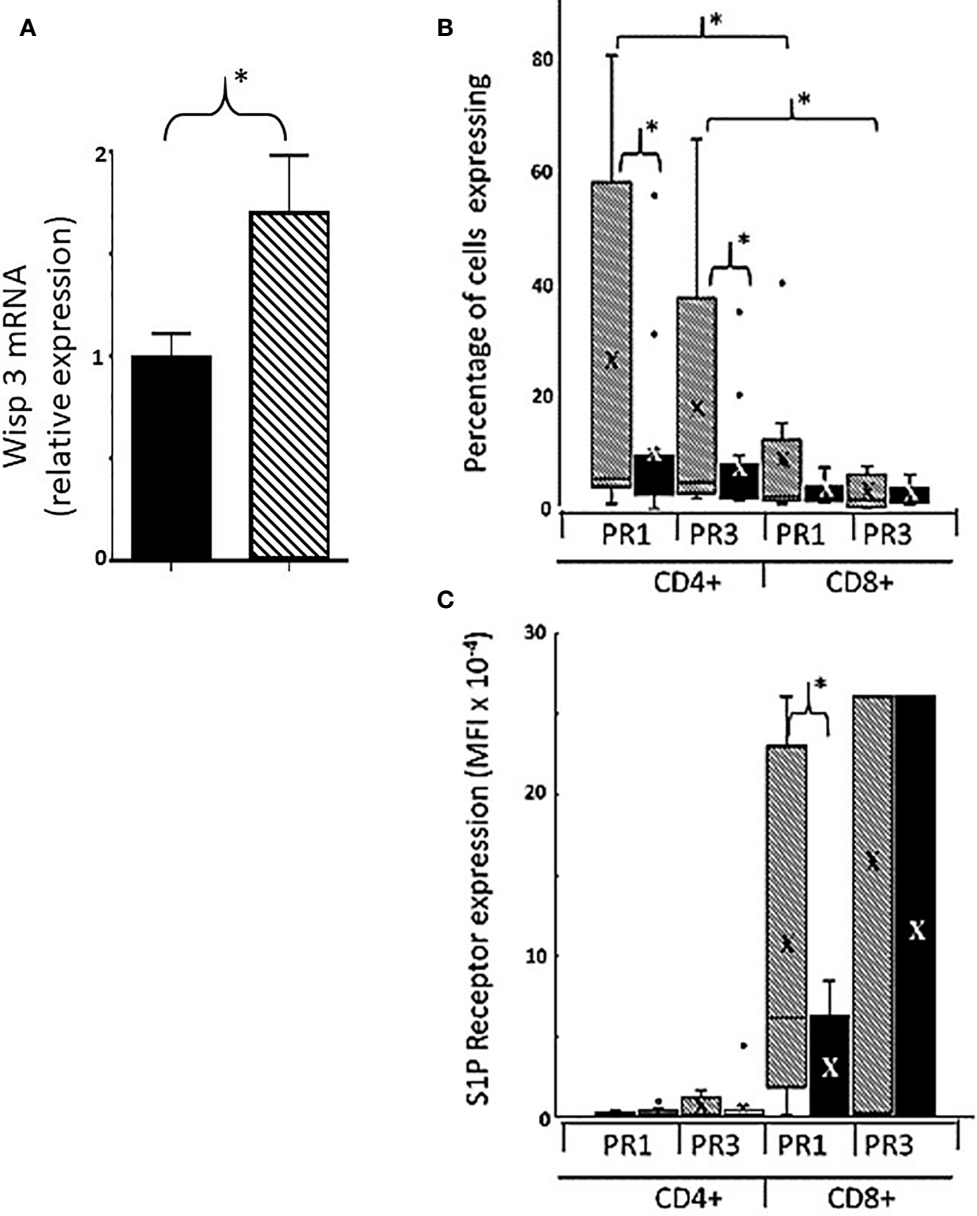
Sexually dimorphic expression of thymic Wisp3 mRNA and Expression of type 1 and 3 receptors for sphingosine-1-phosphate on CD4+ and CD8+ cells from postnatal day 7 FCG pup thymi. RNA from thymic epithelium/stroma of male and female FCG mice at post-natal day 7 was analyzed for Wisp3 mRNA expression by RT-QPCR **(A)**. Data are presented as relative mRNA expression normalized to GAPDH and setting the male value as 1. *n* = 8 males (both XXM and XYM, solid bar) and 8 females (both XXF and XYF, cross-hatched bar). *p < 0.05. Percentage of cells expressing either S1PR1 or S1PR3 **(B)** and mean fluorescence intensity for these receptors **(C)**. Data are shown as box and whisker plots. X= mean. n = 11 females and 13 males. Males includes both XYM and XXM (solid bars) and females includes both XXF and XYF (cross-hatched bars). *p <0.05. Effect size for CD8+PR1 MFI in males versus females **(C)** is 1.48.

Given the early effect of gonadal sex on splenic cell numbers and the maintenance of the sex difference throughout life (compare **Figures 1** and **3**) we considered the possibility that sex had a permanent effect on gene expression in the thymic epithelium/stroma. However, the gonadal sex differences in gene expression were small. Of the 73 genes that displayed statistically significant sex differences in expression, there were only two that exhibited both a fold difference of 0.2 or greater and a probe level expression of 200 or higher (GEO: GSE 135071). These were Glucocorticoid induced transcript 1 (Glcci1) and Wnt1 Inducible signaling pathway protein 3 (Wisp3), and both were expressed higher in females. In a new set of 8 males and 8 females, quantitative RT-PCR did not confirm the difference for Glcci1, but did confirm a sex difference in Wisp3, which was expressed at a 70% higher level in female thymic epithelium/stroma (**Figure 12A**).

Because of the crucial role of sphingosine-1-phosphate (S1P) in thymocyte egress, we also examined expression of proteins that contribute to the S1P chemotactic gradient (38). The amount of S1P is affected by the kinase that produces it and the lyase that breaks it down. Sphingosine-1-phosphate lyase 1 (Sgpl1) is produced by thymic epithelium and dendritic cells (38) and decreases the amount of S1P around the maturing thymocytes relative to the blood (39). The S1P in the blood then has a chemotactic effect on single positive thymocytes ready to leave. Initially, we hypothesized that thymi of gonadal females would express higher levels of the lyase, thereby making egress of thymocytes easier. However, there was no significant difference between FCG gonadal males and females in expression of mRNA for either the kinase or lyase (data not shown).

In order to respond to gradients of S1P, thymocytes express S1P receptors. Although the S1P receptors are a family of five G protein-coupled receptors (40), S1PR1 has been reported to be the most important in the thymus (41). However, S1PR3 has a potential Sry response element and so we considered the possibility that it could also have been of significance to the sex difference. Flow cytometry (see **Supplementary Figure 5** for gating strategy and dot plots) showed that for CD4+ cells, a larger proportion of female *versus* male cells expressed S1PR1 and/or S1PR3 (**Figure 12B**). For CD8+ cells, the mean fluorescence intensity (MFI) for both cell surface receptors was much higher than for CD4+ cells (**Figure 12C**), suggesting greater reliance on the S1P gradient for thymic egress of CD8+ cells. In addition, there was a substantial sex difference in the MFI for S1PR1 on CD8+ cells, with female cells expressing ~4-fold the density of male cells (**Figure 12C**).

## Discussion

### The Experimental Model

We have used the FCG mouse model to determine when measurable sex differences in the immune system first appear and what influences their development. Because our pilot data indicated early appearance of T cell sex differences at a time when T-cell immune responses are immature, we examined sex differences by focusing on relative T cell numbers. We chose the FCG model because we expected to discover effects due to both chromosomal complement and neonatal endocrinology. However, using the measures reported, we saw no chromosomal complement effects and hence have displayed many results as just gonadal male *versus* female to make the many graphs and tables somewhat easier to digest. Our findings using these measures in no way eliminate the possibility that a two X *versus* an X and Y chromosomal complement plays additional roles in sex differences in specific immune responses. Indeed, a chromosomal effect has been reported in the FCG model when examining susceptibility to experimental autoimmune encephalitis, a model for multiple sclerosis (42, 43).

### Basis of the Observed Sex Difference

Our finding that the splenic cell number sex difference was evident in adult animals gonadectomized 4 weeks earlier shows that what we are measuring is not the result of activational, reversible effects of gonadal hormones i.e. that the cell number difference is unlike the effects of gonadal hormones for example on expression of the autoimmune regulator, AIRE (44, 45),. Nevertheless, and as mentioned in the introduction, modulatory and reversible effects of sex steroids on immune responses do occur [e.g (2–5).]. While considering AIRE, it is worth mentioning that there was no gonadal sex-based differential expression in the day 7 neonatal thymi (see microarray data uploaded to GEO: GSE 135071), consistent with the findings of others (44).

Because the sex difference appears by postnatal day 7 and was not influenced by the number and type of sex chromosomes, we were left with two important differences between males and females: the perinatal testosterone surge and expression of *Sry*, which is the only gene by necessity left associated with gonadal maleness in the FCG model.

### Sex Steroids and Thymocyte Development

**Figure 13:**
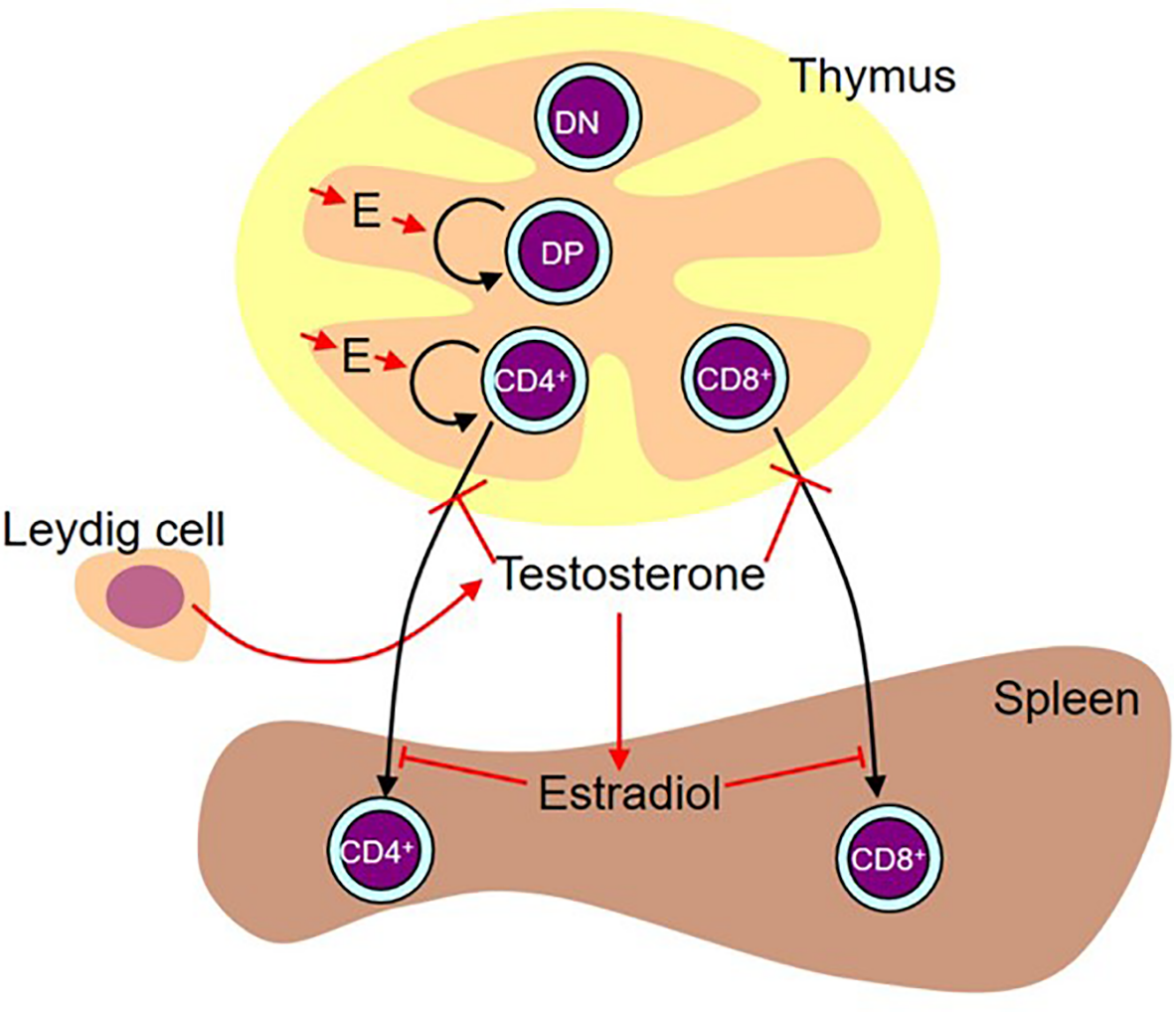
Working diagram illustrating the results. Estradiol (E) produced by the thymic epithelium/stroma stimulates proliferation of double positive (DP) and CD4 single positive cells. While administered testosterone (presumably aromatized to estradiol) and estradiol also had a stimulatory effect on double negative (DN) and CD8 single positive cell number, we are basing the preference for stimulation of the pathway leading to the production of CD4+ cells on the flutamide and letrozole results on the assumption that these results are more likely representative of the effect of physiological levels of locally produced steroids. In males, there is a peripartum surge of testosterone produced by the Leydig cells in the testes. This testosterone inhibits release of mature cells from the thymus. This activity is testosterone-specific (i.e. it is not aromatized). In the spleen, some of this testosterone is aromatized to estradiol, which in turn inhibits T cell seeding of the spleen.

Concurrent analysis of both sexes proved invaluable to dissection of the effects of the perinatal surge in testosterone and eventual production of the model shown in **Figure 13**. Without analysis in both sexes, we would not have been able to unravel the differential effects of the sex steroids in the thymus and spleen and we would have been completely misled by the SiRNA knockdown of Sry.

Since there is no peripartum surge in gonadal testosterone in females and yet the testosterone antagonist, flutamide, and the aromatase inhibitor, letrozole, had equivalent effects on both Ki67 and Bax expression in male and female thymocytes, we conclude that there is some other source of these hormones available to influence thymocyte development, regardless of sex. At least based on microarray analysis of gene expression, this alternate source could be the thymic epithelium/stroma. The approximate equivalence of flutamide and letrozole and testosterone and estradiol on intrathymic parameters also shows that testosterone needs to be aromatized to estradiol to exert this effect. The microarray analysis further showed that the thymic epithelium/stroma expresses all necessary enzymes for the aromatization of locally produced testosterone in both sexes. While we cannot exclude an alternate non-gonadal source of the hormones and indirect effects, there are no measurable amounts in the circulation of female neonates (22). Therefore, production within the thymus where hormone effects can be integrated with other micro-environmental cues seems most likely. Also, although expression of the enzymes does not necessarily translate to activity, others have at least demonstrated activity of the first part of the biosynthetic pathway by measuring pregnenolone accumulation after inhibition of later steps in steroidogenesis (46, 47). With demonstrated expression of the rest of the necessary enzymes, continuation to the production of estradiol seems likely.

The effects of flutamide and letrozole on Bax and Ki67 were initially a great puzzle since in most tissues, one would normally expect opposite effects of treatments on the expression of Bax and Ki67. However, in thymocytes, both drugs decreased the percentage of cells expressing Bax, which might be expected to increase cell number, as well as decreasing the percentage expressing Ki67, which might be expected to decrease cell number. Given that the Ki67 results are supported by the administration of testosterone (and estradiol) which increased cell number, we assume that Ki67 more accurately reflects what is happening and that Bax expression is not necessarily indicative of a cell undergoing apoptosis. In this regard, it is interesting that we observed concurrence of Bax and Ki67 expression, a phenomenon that has been previously described by others (32) thereby suggesting that Bax expression per se is not directly indicative of apoptosis. In addition, an approximately 60% reduction in Bax+ cells had no effect on cell number. Bax had seemed like a good choice of apoptotic marker given the literature on its important role in apoptosis of double positive thymocytes (48), but clearly the regulation of apoptosis, especially during negative selection, is more complex (49).

Administration of testosterone or estradiol increased the number of cells in the thymus. While the effect of testosterone to increase thymocyte number could have been secondary to inhibition of release of mature single positive cells, this could not be the explanation for the increase in thymocytes with estradiol since there was in this case no significant effect on the number of single positive cells in the thymus. We deduce from the reduced number of Ki67+ cells with aromatase inhibitor that endogenous thymic estradiol has a positive influence on the proliferation of thymocytes in the neonate, more specifically on the number of double positive and single positive CD4 cells (**Table 1B**). This is a heretofore unrecognized role for estradiol in pre-pubertal thymic physiology (illustrated in **Figure 13**). Given the rejuvenating effects of gonadal sex steroid ablation on older thymi [reviewed in (50)], this was an unexpected finding. However, there is ample precedent for opposite effects of gonadal steroids in neonates *versus* mature animals [e.g (51)].

### Sex Steroids and Thymic Egress

The finding of an unexpected, non-gonadal source of sex steroid that impacts thymocyte development complicated interpretation of results with administration of both flutamide and letrozole, but administration of testosterone and estradiol to both sexes helped to sort through the possible interpretations. Once thymocytes are mature single positive cells, they would normally exit the thymus. Since the thymic content of single positive cells increased in response to testosterone, but not estradiol, in both sexes, the results support a role for testosterone in inhibiting either the final maturation of single positive cells in the thymic medulla (52) or the movement of cells through the emigration barriers of thymic epithelium and vessel endothelium and associated basal laminae (53).

### Sex Steroids and Splenic Seeding

Although administration of testosterone to males and females showed the same effect in the thymus and testosterone reduced splenic seeding in males, there was not an equivalent reduction in splenic T cells in response to testosterone in females. Therefore, we considered the additional existence of a sex difference in post-thymic distribution of T cells, especially as we could find no evidence of differential proliferation of T cells once in the spleen between the sexes. Estradiol decreased splenic content of T cells in females, a result consistent with the effects of neonatal letrozole to increase CD4+ T cells in the male spleen. In other words, following the perinatal surge of testosterone comes inhibition of release of T cells from the thymus and reduced seeding to the spleen, with the former dependent on testosterone itself and the latter requiring aromatization of testosterone to estradiol (illustrated in **Figure 13**). Making the assumption that mRNA for the aromatase is reflective of relative enzyme activity, the gonadal male spleen has a superior ability to aromatize testosterone and therefore to block T cell seeding to the spleen. Furthermore, the neonatal male spleen is the only one exposed to a source of circulating testosterone to serve as substrate for aromatization.

### Organizational Effects

The degree of sex difference in splenic cell number is the same at 7 days of age as in gonadectomized adults. This implies that the consequences of the perinatal surge in testosterone and *Sry* expression on thymic release and splenic seeding are irreversible and organizational in nature and likely related to epigenetic changes. For thymic epithelium/stroma, there were surprisingly few differentially expressed genes, although females expressed Wisp3 at a 70% higher level. Since thymic function has not been examined in Wisp3 transgenic or knockout mouse models, we can only speculate at this time about what this might mean. However, Wisp3 is a member of the CCN (CTGF, CYR61, and NOV) family of connective tissue growth factors. Secreted CCNs regulate cell proliferation, survival, migration, adhesion, differentiation, and extracellular matrix formation (54–58), and hence Wisp3 could have multiple influences during T cell maturation in the thymus. For example, Wisp3 binds to and inhibits bone morphogenic protein (BMP) (57, 58) and, as mentioned earlier, BMP4 inhibits thymocyte proliferation and progression from the double negative to the double positive stage (37). Inhibition of an inhibitor of thymocyte maturation would permit eventual increased output of single positive T-cells in females. Additionally, Wisp3 may affect extracellular matrix (54–56) and therefore both migration within, and emigration from, the thymus. Interestingly, the Wisp3 promoter has a potential Sry/Sox response element at -383, which could participate in repression of Wisp3 expression in gonadal males. For splenic seeding inhibited by estradiol, long-lasting effects may include aromatase expression at about double-triple in males *versus* females. Finally, we have the sex difference in thymocyte expression of the S1PRs. Given that CD4+ and CD8+ cells derive from CD4CD8 double positive cells, sex specific expression of S1PRs must be influenced during differentiation to the single positive stage. Comparing gonadal females and gonadal males, a greater percentage of CD4+ cells expressed S1PRs in females and the number of S1PR1 receptors per CD8+ cell was greater. Both of these findings would be expected to enhance T cell thymic egress in females and hence produce a greater potential for splenic seeding. Conversely, a reduced percentage of cells expressing S1PRs and CD8+ cells with a lower expression of S1PR1 in males would be expected to reduce thymic egress and therefore the potential for splenic seeding. In addition, reduced expression of these receptors in males may dictate the destination of many of those released from the thymus; low expression of S1PR1 is characteristic of localization to non-lymphoid tissues (59). It is possible therefore that in addition to what we have uncovered in regard to splenic seeding, sex differences in the seeding of peripheral tissues may contribute to the numbers of CD8+ cells present or not present in the spleen.

### Origin of the S1PR1 Sex Difference

The working model provided as **Figure 13** shows what we have been able to deduce about hormonal effects on the thymus and spleen for both CD4+ and CD8+ T cells but does not account for the much greater sex difference seen for CD8+ cells. Given a) the demonstration that the CD8+ difference is not brought about by the perinatal surge of testosterone in males, b) the fact that the only other difference between both male and both female genotypes is the expression of Sry, and c) with the exception of Wisp3, the minor differences in thymic epithelial expression between the sexes, it seems most likely that the sex difference is an effect of Sry on S1PR1 expression within the CD8+ cell itself once committed to this differentiation. However, in the absence of a known Sry response element in the S1PR1 gene, this would presumably be an indirect effect, possibly a consequence of reduced Wisp3. This hypothesis is an obvious avenue for future investigation.

### Limitations of the Model

It is known that there are multiple copies of *Sry* in the FCG model (22), and Sry mRNA is expressed at a higher level in FCG males than in WT males, at least in brain (60). Moreover, the sex difference in terms of splenic cell number is exaggerated in this model (unpublished observation). The benefits of an exaggerated model include a greater ability to detect changes in response to experimental manipulation, but the dangers are, of course, that overexpression of Sry could introduce factors not representative of normal physiology. However, although those caveats may be relevant for some of the results, at least two major conclusions of the current study are not related to the FCG model: first is the intrathymic use of sex steroids, which occurs in both males and females, including the XX females, which are entirely normal. The second is the impact of the testosterone surge in males since this occurs in both FCG and wildtype males.

### Immune Significance

While numbers of total CD4+ and CD8+ cells in the spleen do not directly correlate with differences in peripheral immune responses, having more CD4+and CD8+ cells in female spleens allows for a higher level of antigen screening of the blood and subsequent T cell proliferation and distribution of effector cells to peripheral non-lymphoid tissues in response to infection (61). Also, of note in this regard was the greater proportion of potential effector T cells to Tregs in females, which statistically could increase the chance of more profound immune responses.

We conclude that the work has uncovered early development of sex differences in the immune system and previously unrecognized roles for steroid hormones in the regulation of cell development and egress in the neonatal thymus and in the seeding of T cells to the spleen. In addition, sex differences in CD8+ cell numbers and distribution are likely the result of an indirect effect of Sry on expression of S1PR1 receptors.

## Data Availability Statement

Datasets containing individual animal values will be made available upon request to the corresponding author. Microarray data have been uploaded to GEO: GSE 135071.

## Ethics Statement

The animal study was reviewed and approved by University of California, Riverside Institutional Animal Care and Use Committee, AUP A20160040E.

## Author Contributions

Experimentally. MG and K-hC conducted the most important experiments that appear in the final manuscript. RD-G conducted the thymic microarray and SiRNA studies. LM conducted many pilot studies and contributed data for **Figures 1** and **2**. TY measured testosterone levels in the FCG males. YI analyzed the microarray data. LR maintained the FCG colony and genotyped most of the FCG pups. KR examined the anogenital distances. QW examined splenic aromatase expression. and AW, HM, and AA directed the study. All authors contributed to the article and approved the submitted version.

## Funding

This work was supported by a grant from the Eunice Kennedy Shriver National Institute for Child Health and Human Development # 65099 to AW. This agency played no part in design of the study, collection, analysis, and interpretation of data, nor in writing the manuscript.

## Conflict of Interest

The authors declare that the research was conducted in the absence of any commercial or financial relationships that could be construed as a potential conflict of interest.
