# Supplementary figures and images for: Sex Differences in the Immune System Become Evident in the Perinatal Period in the Four Core Genotypes Mouse
Source: Front Endocrinol (Lausanne). 2021 May 27;12:582614. doi: 10.3389/fendo.2021.582614 (PMC8191418; doi:10.3389/fendo.2021.582614)

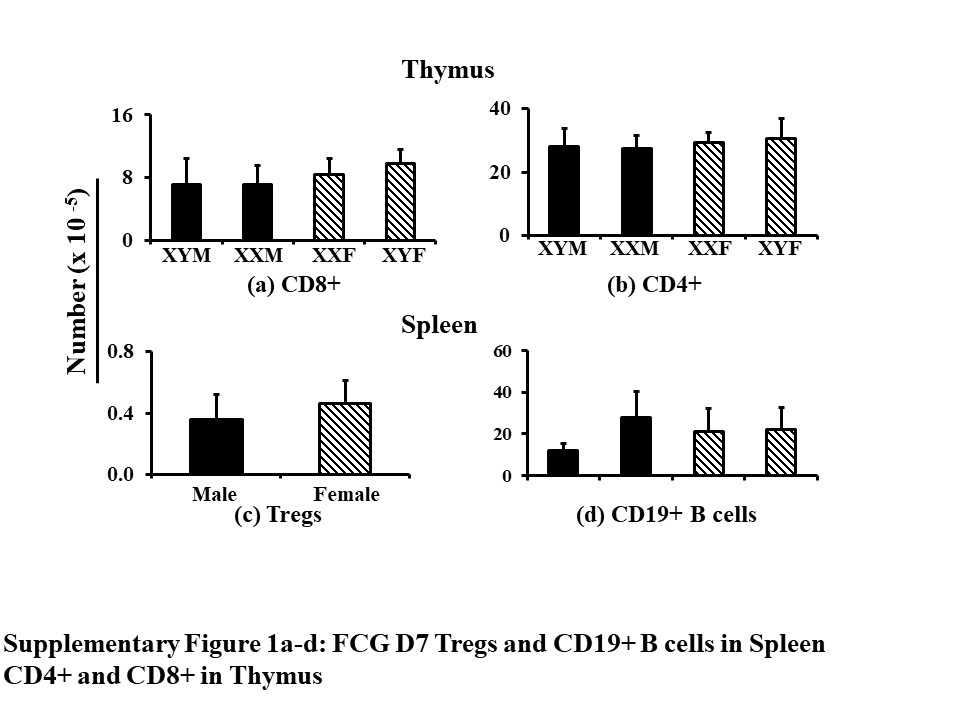

Supplement: Supplementary file 1 [file Image_1.jpeg]

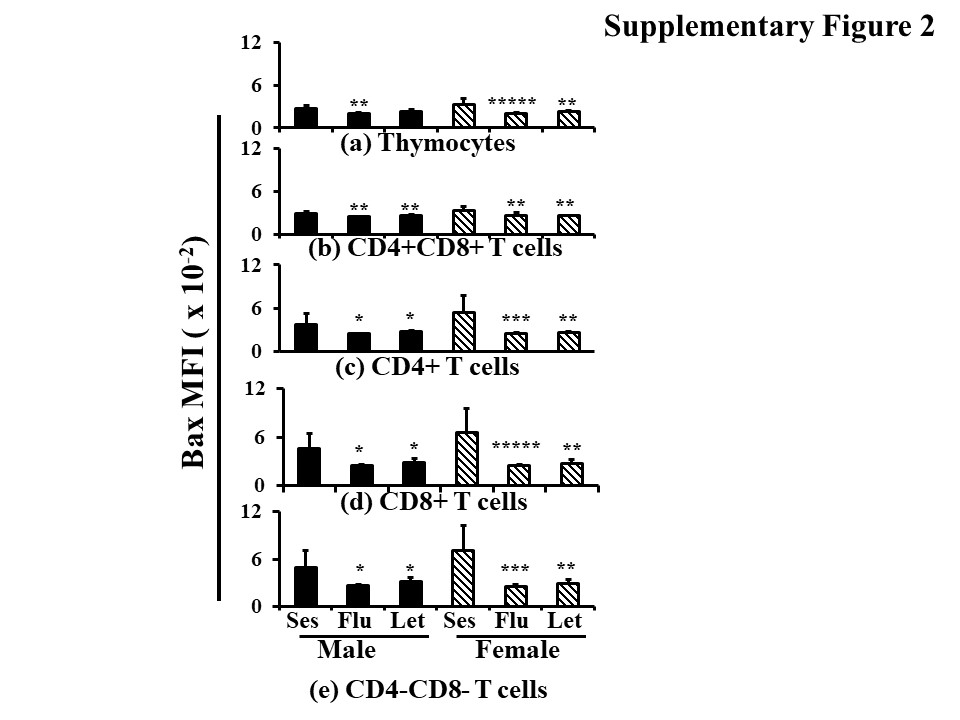

Supplement: Supplementary file 2 [file Image_2.jpeg]

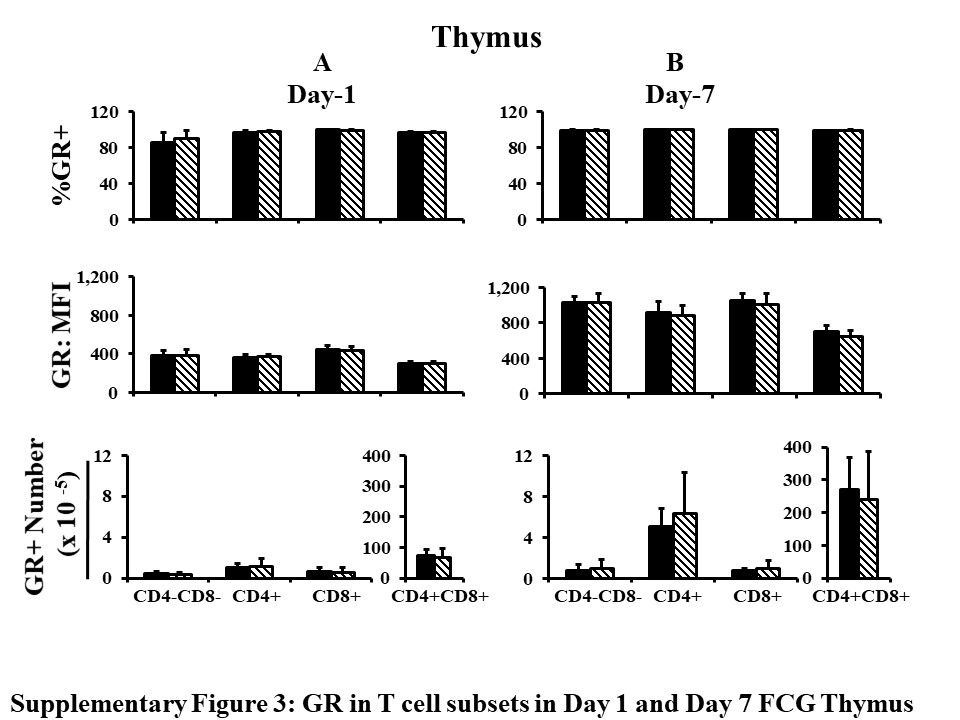

Supplement: Supplementary file 3 [file Image_3.jpeg]

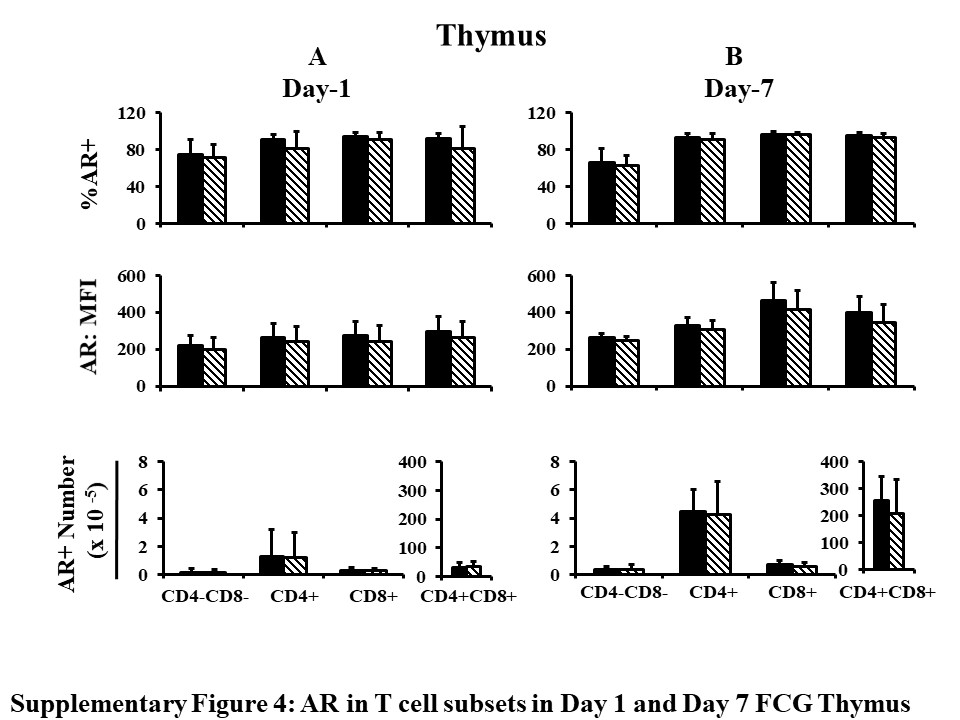

Supplement: Supplementary file 4 [file Image_4.jpeg]

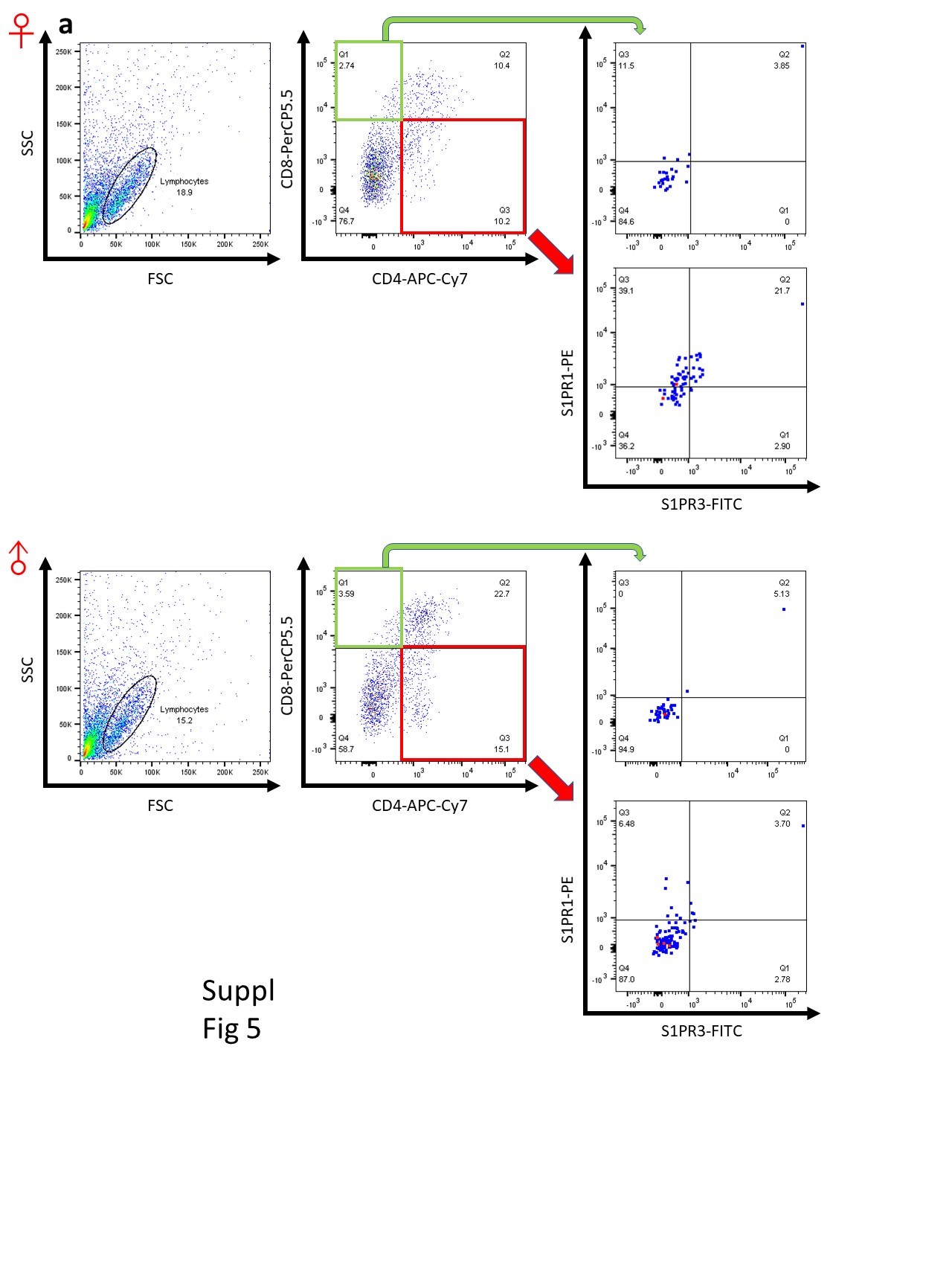

Supplement: Supplementary file 5 [file Image_5.jpeg]
