# Supplementary material for: Sex Differences in the Immune System Become Evident in the Perinatal Period in the Four Core Genotypes Mouse
Source: Front Endocrinol (Lausanne). 2021 May 27;12:582614. doi: 10.3389/fendo.2021.582614 (PMC8191418; doi:10.3389/fendo.2021.582614)
Supplement: Supplementary file 6 [file DataSheet_1.docx]

| Male  day 1 | Female day 1 |  | XYM  5 months | XXM  5 months | XXF  5 months | XYF  5 months |
| --- | --- | --- | --- | --- | --- | --- |
| 1.83 ± 1.06 | 0.67 ± 0.1 |  | 2.7 ± 0.43 | 2.2 ± 0.24 | 0.11 ± 0.04 | 0.16 ± 0.03 |

Supplementary table 1: Testosterone levels in males and females at day 1 and all genotypes at 5 months of age. Values are in ng/ml ± SD. Male genotypes not significantly different from each other, female genotypes not significantly different from each other. Each male genotype significantly different from each female genotype (*p*<3 x 10^-5^)
