# Supplementary material for: Sex Differences in the Immune System Become Evident in the Perinatal Period in the Four Core Genotypes Mouse
Source: Front Endocrinol (Lausanne). 2021 May 27;12:582614. doi: 10.3389/fendo.2021.582614 (PMC8191418; doi:10.3389/fendo.2021.582614)
Supplement: Supplementary file 7 [file DataSheet_2.docx]

Supplementary Table 2. Relative expression of the listed genes in thymic epithelium/stroma in all males *versus* all females.

|  | Average Signal |  |
| --- | --- | --- |
| Gene ID | Male | Female |
|  |  |  |
| CYP19A1 | 79.3 ± 9.2 | 78.3 ± 1.9 |
| AR | 172.4 ± 29.6 | 182.3 ± 18.3 |
| CYP11A1 | 116.6 ± 6.7 | 110.5 ± 5.3 |
| CYP11B1 | 102.3 ± 11.5 | 97.8 ± 5.5 |
|  |  |  |
| 17 B hydroxysteroid dehydrogenase variants |  |  |
| Hsd17b1 | 124.7 ± 13.5 | 126.4 ± 8.8 |
| Hsd17b2 | 112.1 ± 13.3 | 113.7 ± 12.1 |
| Hsd17b3# | 41.9 ± 2.5 | 45.7 ± 3.3 |
| Hsd17b4 | 760.5 ± 48.0 | 728.6 ± 45.9 |
| Hsd17b6 | 68.0 ± 7.2 | 68.6 ± 4.4 |
| Hsd17b7 | 364.9 ± 59.9 | 368.1 ± 53.3 |
| Hsd17b10 | 955.2 ± 56.1 | 919.7 ± 81.3 |
| Hsd17b11 | 777.7 ± 57.6 | 743.3 ± 41.8 |
| Hsd17b12 | 635.5 ± 73.7 | 603.0 ± 59.2 |
| Hsd17b13 | 71.5 ± 6.9 | 67.5 ± 3.4 |
| Hsd17b14 | 222.4 ± 19.6 | 216.9 ± 17.3 |

There were no significant differences.

#, Below reliable detection.
